# Biosynthesis of Chiral Amino Alcohols via an Engineered Amine Dehydrogenase in *E. coli*

**DOI:** 10.3389/fbioe.2021.778584

**Published:** 2022-01-05

**Authors:** Feifei Tong, Zongmin Qin, Hongyue Wang, Yingying Jiang, Junkuan Li, Hui Ming, Ge Qu, Yazhong Xiao, Zhoutong Sun

**Affiliations:** ^1^ School of Life Sciences, Anhui University, Hefei, China; ^2^ Tianjin Institute of Industrial Biotechnology, Chinese Academy of Sciences, Tianjin, China; ^3^ Department of Chemistry, School of Science, Tianjin University, Tianjin, China; ^4^ Department of Life Sciences and Medicine, University of Science and Technology of China, Hefei, China; ^5^ National Technology Innovation Center of Synthetic Biology, Tianjin, China

**Keywords:** biosynthesis, protein engineering, directed evolution, amine dehydrogenase, chiral amino alcohol

## Abstract

Chiral amino alcohols are prevalent synthons in pharmaceuticals and synthetic bioactive compounds. The efficient synthesis of chiral amino alcohols using ammonia as the sole amino donor under mild conditions is highly desired and challenging in organic chemistry and biotechnology. Our previous work explored a panel of engineered amine dehydrogenases (AmDHs) derived from amino acid dehydrogenase (AADH), enabling the one-step synthesis of chiral amino alcohols via the asymmetric reductive amination of α-hydroxy ketones. Although the AmDH-directed asymmetric reduction is in a high stereoselective manner, the activity is yet fully excavated. Herein, an engineered AmDH derived from a leucine dehydrogenase from *Sporosarcina psychrophila* (*Sp*AmDH) was recruited as the starting enzyme, and the combinatorial active-site saturation test/iterative saturation mutagenesis (CAST/ISM) strategy was applied to improve the activity. After three rounds of mutagenesis in an iterative fashion, the best variant wh84 was obtained and proved to be effective in the asymmetric reductive amination of 1-hydroxy-2-butanone with 4-fold improvements in *k*_*cat*_/*K*_*m*_ and total turnover number (TTN) values compared to those of the starting enzyme, while maintaining high enantioselectivity (*ee* >99%) and thermostability (*T*_*50*_^*15*^ >53°C). In preparative-scale reaction, the conversion of 100 and 200 mM 1-hydroxy-2-butanone catalyzed by wh84 was up to 91–99%. Insights into the source of an enhanced activity were gained by the computational analysis. Our work expands the catalytic repertoire and toolbox of AmDHs.

## Introduction

As essential structural moieties, chiral α-amino alcohols are widely applied to produce synthetic and natural bioactive molecules (Ager et al., 1996; Erlanson et al., 2011). For instance, many pharmaceuticals consist of α-amino alcohols that serve as chiral building blocks (Supplementary Scheme S1). Direct asymmetric reductive amination of ketones with free ammonia to produce chiral amino alcohols is a highly aspirational transformation (Abrahamson et al., 2013; Pushpanath et al., 2017). In the traditional chemical synthesis, it mainly relies on stoichiometric amounts of chemical reducing agents or organometallic catalysts, which are limited by low stereoselectivities, the formation of the alcohol as side product, and the requirement of extreme reaction conditions (Larrow et al., 1996; Breuer et al., 2004; Ma et al., 2010; Nugent and El-Shazly, 2010; Karjalainen and Koskinen, 2012; Xie et al., 2020; Hollmann et al., 2021). Alternatively, enzymes as catalysts are increasingly explored as essential tools in asymmetric reductive aminations (Reetz, 2011; Abrahamson et al., 2013; Schrittwieser et al., 2015; Chen and Arnold, 2020; Winkler et al., 2021). In recent years, a number of enzymes have been identified that are capable of catalyzing the asymmetric reductive amination of ketones (Wu et al., 2021), including lipases (Francalanci et al., 1987), acylases (Wang et al., 2016), transaminases (Wu et al., 2017), and imine reductases (Matzel et al., 2017). Moreover, native amine dehydrogenases (AmDHs) have been identified that they can directly utilize ammonia as a sole amino source in reductive amination but with insufficient enantioselectivity (Itoh et al., 2000; Mayol et al., 2019).

**SCHEME 1:**
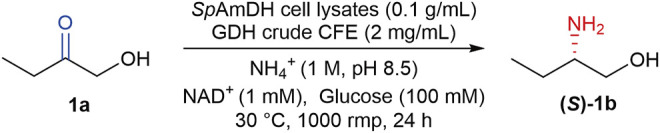
*Sp*AmDH-catalyzed asymmetric reductive amination of 1a to (*S*)-1b by using ammonia as the sole amino donor, and glucose dehydrogenase (GDH) cell-free extract (CFE) for NADH cofactor regeneration.

Apart from the native AmDHs, the Bommarius group at Georgia Tech has engineered two natural amino acid dehydrogenases (AADHs), including a leucine dehydrogenase from *Bacillus stearothermophilus* (Abrahamson et al., 2012) and a phenylalanine dehydrogenase from *Bacillus badius* (Abrahamson et al., 2013). After altering two determinant residues of carboxylate recognition, the natural AADHs were transformed to AmDHs, thereby eliminating the activity toward ketone acids while affording new activity toward ketones. Taking this advantage, more natural AADHs from diverse organisms have been explored and engineered to AmDHs based on the introduction of two-point mutations, which were then harnessed in the asymmetric production of chiral amines (Au et al., 2014; Chen et al., 2015; Ye et al., 2015; Franklin et al., 2020; Liu et al., 2020). In addition, the engineered AmDHs derived from AADHs were also utilized in the preparation of chiral amino alcohols, which are usually with very high enantioselectivity (>99% *ee*), making these enzymes of potential value in biocatalysis (Chen et al., 2019). Our previous work has characterized and engineered five novel AmDHs from natural AADHs by genome mining, these newly identified AmDHs provided reductive amination of a broad range of prochiral α- and β-hydroxy ketones in a high stereoselective manner (Wang et al., 2020). As an example, the engineered AmDH derived from the leucine dehydrogenase from *Sporosarcina psychrophila* (*Sp*AmDH), enabled the reduction of 1-hydroxybutan-2-one (1a) to (*S*)-2-aminobutan-1-ol ((*S*)-1b, Scheme 1) with >99% selectivity, while the conversion is modest (∼60%) at a substrate concentration of 50 mM (Wang et al., 2020).

In this work, we sought to optimize the activity of *Sp*AmDH in the biosynthesis of (*S*)-1b, which is an important intermediate desired in the preparation of antitubercular drugs ethambutol (Pablos-Méndez et al., 1998; Supplementary Scheme S1). When aiming at the improvement of activity and/or selectivity, the combinatorial active-site saturation test (CAST) combined with iterative saturation mutagenesis (ISM) has been emerged as a powerful means in protein engineering (Reetz et al., 2005; Yanai et al., 2013; Qu et al., 2020; Li D. et al., 2021; Li J. et al., 2021; Qu et al., 2021; Zheng et al., 2021). Taking the advantage of CAST/ISM, three robust variants of *Sp*AmDH with improved activity and high stereoselectivity were obtained, and their potential as biocatalysts in the preparative scale reactions was explored. Computational docking simulations were also performed to rationalize the elevated activity of *Sp*AmDH variants.

## Materials and Methods

### Chemicals and Reagents

Hydroxy ketones, chiral amino alcohols, and other chemical reagents were purchased from Bidepharm (Shanghai, China), Energy Chemical (Shanghai, China), Kaiwei Chemical (Shanghai, China), Acmec (Shanghai, China), Arkpharm (Chicago, United States), Accela (Shanghai, China), Aladdin (Shanghai, China), Macklin (Shanghai, China), Heowns (Tianjin, China), and CINC (Shanghai, China). PrimeSTAR DNA polymerase and restriction enzyme Dpn I were ordered from TAKARA and NEB, respectively. The primer synthesis and sequencing were carried out by GENEWIZ. A plasmid preparation kit was purchased from TIANGEN Biotech. All other chemical reagents can be obtained through commercialization, unless otherwise noted.

### Site-Directed Mutagenesis

Mutagenesis was constructed by using the overlap PCR and megaprimer approach (Tyagi et al., 2004) with high-fidelity master mix polymerase. Reaction mixtures (50 μl) typically contained ddH_2_O (22 μl), 2× high-fidelity master mix polymerase (25 μl), template DNA (1 μl, 50 ng), forward primer (1 μl, 0.2 μM), and reverse primer (1 μl, 0.2 μM). The PCR conditions for short fragment were as follows: 98°C, 2 min (98°C, 10 s; 55°C, 15 s; 72°C, 30 s) 30 cycles; 72°C, 3 min. For mega-PCR (Tyagi et al., 2004), 1st PCR product (2 μl, 800 ng) was used as primer, and the PCR conditions as below: 98°C, 2 min (98°C, 10 s; 60°C, 15 s; 72°C, 3.5 min) 30 cycles, 72°C, 5 min. The PCR products were treated with restriction endonuclease Dpn I for 3 h and then electroporated into *E. coli* BL21 (DE3). After culturing for 12 h, the colonies on the plate were washed with ddH_2_O, and the plasmids were extracted and sequenced. The primers used for constructing single-site saturation mutagenesis and combinatory saturation libraries were listed in Supplementary Tables S1, S2, respectively.

### Screening of Saturation Mutagenesis Libraries

Clones from the plate were transferred to 96-well deep-well culture plates containing 300 μl of LB medium (50 μg/ml kanamycin), shaking at 37°C and 800 rpm for 10 h. Then 120 μl of the aforementioned culture broth was transferred to a 96-well glycerol plate, 60 μl of glycerol (60%, v/v) was added, and stored at −80°C. At the same time, 800 μl of the TB medium was added to the 96-well deep-well culture plates, IPTG (0.2 mM) and kanamycin (50 μg/ml) were added, and the culture was shaken at 30°C, 800 rpm for 12 h for protein expression. The cells were collected by centrifugation at 4,000 rpm, 4°C for 10 min. The cells were washed and resuspended with 400 μl of potassium phosphate buffer (PBK, 50 mM, pH 7.4). DNase I (6 U/ml) and lysozyme (1 mg/ml) were added to the culture and were shaken at 30°C for 1 h to lysis cells. After low-temperature centrifugation (4°C, 4,000 rpm, for 30 min), the supernatant was used for enzyme activity determination. The reductive amination reaction was performed in NH_4_Cl/NH_3_·H_2_O buffer (1 M, pH 8.5) containing 200 μl supernatant, 10 mM substrate 1a, 1 mM NAD^+^, 100 mM glucose, and 2 mg/ml GDH at 30°C, 800 rpm for 24 h. After that, 1 μl of 1M para-methoxy-2-amino benzamidoxime (PMA) was added to 99 μl of the reaction solution, and fluorescence was measured using an excitation wavelength of 380 nm and emission wavelength of 520 nm for substrate consumption (Mei et al., 2020).

### Active Assay

For single point saturation mutation library rescreening, the best clones from 96-well glycerol plates were cultivated, expressed in a shake flask and used for biotransformation. Reductive amination reactions were performed in a reaction mixture (0.5 ml) containing 1 M NH_4_Cl/NH_3_·H_2_O buffer (pH 8.5), 1 mM NAD^+^, 100 mM glucose, 2 mg/ml GDH cell-free extract (CFE), 6 U/ml DNase I, 20 mM 1a, and 20 mg/ml mutant CFE in 2 ml Eppendorf tubes at 30°C, 1,000 rpm for 24 h in a thermostatic metal bath. For the combinatorial saturation mutation library screening, using the same procedure as before, except for that, the concentration of 1a and the wet cell of mutant were 40 mM and 0.1 g/ml, respectively. After the reaction was over, the aforementioned reaction solution was boiled for 5 min and then centrifuged at 12,000 rpm for 10 min to remove the precipitate. The supernatant was measured by HPLC to detect the conversion of 1a. The conversions of hydroxy ketones substrates (1a–8a) to chiral amino alcohol products (1b-8b) were measured with Marfey’s reagent (1-fluoro-2, 4-dinitrophenyl-5-L-alanine amide) for pre-column derivatization. The reaction mixture was mixed with 100 μl sample, 30 μl of Marfey’s reagent (14 mM), 80 μl NaHCO_3_ (1 M), and 200 μl DMSO at 80°C, 1,000 rpm for 10 min. Finally, 10 μl HCl (4 M) was added to stop the reaction. Detection conditions were given as follows: Zorbax SB-C18 column (4.6 × 150 mm, 5 μm), detection wavelength: 340 nm, temperature: 25°C, flow rate: 1 ml/min, loading volume: 10 μl, mobile phase buffer A: ddH_2_O (0.1% trifluoroacetic acid), buffer B: methanol (0.1% trifluoroacetic acid), gradient program: 40% B, hold for 6 min, increase B to 60% in 9 min, hold for 3 min, decrease B to 40% in 2 min, and hold for 5 min. More details are listed in Supplementary Table S3.

### Protein Expression and Purification

*E. coli* BL21(DE3) glycerol bacteria containing *Sp*AmDH gene were cultivated in 5 ml LB liquid medium (50 μg/ml kanamycin) for 10 h. The previous culture was transferred to the TB medium (100 ml) (50 μg/ml kanamycin) and was cultured at 37°C, 220 rpm. The culture was induced by the addition of IPTG (0.1 mM) when OD_600_ reached 0.8 and was then allowed to grow for an additional 12 h at 20°C. The cells expressing AmDHs were harvested, sonicated, and centrifuged (4°C, 12,000 rpm) for 60 min to remove the precipitate. The supernatant with soluble His-tagged protein was filtered using a 0.45-µm filter membrane and was verified by SDS-PAGE analysis (Supplementary Figure S1). The column (HisTrap FF, 5 ml) was washed with A buffer (50 mM PBK containing 300 mM NaCl and 20 mM imidazole, pH 8.0) before and after the supernatant was loaded. The proteins were eluted with B buffer containing a high concentration of salt (50 mM PBK containing 300 mM NaCl and 500 mM imidazole, pH 8.0). The eluates were ultrafiltered (4°C, 3,500 rpm) with an ultrafiltration tube (10,000 Da) to concentrate and replace the buffer (25 mM PBK, 100 mM NaCl, 5% glycerol, pH 8.0). The protein concentration was confirmed by measuring the absorbance at 280 nm using a Nano-300 micro-spectrophotometer.

### Total Turnover Numbers Assay for Conversion of Substrate 1a to 1b Using *Sp*AmDH Variants

The asymmetric reductive amination reactions were performed with 0.32–0.64 mg/ml purified enzyme, 1 M NH_4_Cl/NH_3_·H_2_O buffer (pH 8.5), 1 mM NAD^+^, 100 mM glucose, 2 mg/ml GDH CFE, and 40 mM 1a. The reaction mixture was proceeded at 30°C, 1,000 rpm for 24 h. The product was then detected by HPLC. TTN was defined as the molar number of the product yield divided by the catalyst concentration (Qu et al., 2019).

### Determination of Kinetic Parameters and Thermostability

The kinetic parameters were obtained by measuring the initial velocities of NADH consumption (the initial rate of change in absorbance at 340 nm) in the enzymatic reaction and fitting the curve according to the Michaelis–Menten equation (Supplementary Figure S2). The activity assay was performed in a mixture containing 0.2 mM NADH, 1–30 mM 1-hydroxy-2-butanone, 1 M NH_4_Cl/NH_3_·H_2_O (pH 8.5), and the purified enzyme (1 mg/ml). The reaction was initiated by the addition of the enzyme and was monitored for 5 min at 30°C. The activity was determined by measuring NADH oxidation from a decrease in the absorbance at 340 nm (ε = 6,220 M^−1^ cm^−1^) and then Origin was used to perform the nonlinear fitting of the Michaelis–Menten equation. One unit (1 U) of activity is defined as the amount of enzyme required to consume 1 μM NADH in 1 minute. For thermostability, 1 mg/ml pure enzyme solution was incubated at different temperatures (30–70°C) for 15 min, followed by measuring the residual activity in 1 M NH_4_Cl/NH_3_·H_2_O (pH 8.5) containing 0.2 mM NADH, 10 mM substrate 1a at 50°C for 2 min. All experiments were conducted in triplicate.

### Preparative-Scale Reactions Using *Sp*AmDH Mutants

The *Sp*AmDH mutants wh18 and wh84 were inoculated in the 5 ml LB medium (50 μg/ml kanamycin) for 10 h (37°C, 220 rpm). The aforementioned culture was inoculated into the 1,000 ml TB medium (50 μg/ml kanamycin) and cultured at 37°C, 220 rpm until the OD_600_ reached 0.8–0.9. IPTG was then added to a final concentration of 0.1 mM, and the culture was allowed to grow for additional 16 h at 20°C, 220 rpm. The cells were pelleted by centrifugation for 20 min (4°C, 4,000 rpm) and washed once with phosphate buffer (50 mM, pH 7.4). Subsequently, 0.1 g/ml wet cells, 1 M NH_4_Cl/NH_3_·H_2_O buffer (pH 8.5), 1 mM NAD^+^, 100 mM glucose, 2 mg/ml GDH CFE, 6 U/ml DNase I, 1 mg/ml lysozyme, and 1a (100, 200 mM) were mixed for 10 ml in a Erlenmeyer flask. The reaction was performed at 30°C, 220 rpm for 24 h in a shaker with triple replica. Then 200 μl samples were taken at 0, 1, 2, 3, 4, 8, 12, 18, and 24 h and then prepared and analyzed by HPLC. The 100 mM scaled-up reaction of 1a catalyzed by wh84 was terminated by adding 5% H_2_SO_4_ to pH <2 and then centrifuged at an rpm of 4,000 for 20 min at 4°C, to collect the supernatant. The product ((*S*)-2a) was purified via an ion exchange method with Dowex® 50WX8 ion exchange resin (Chen et al., 2015). The column was prepared by washing with 100 ml ddH_2_O and 50 ml 5% w/v H_2_SO_4_. Then, the acidified reaction supernatant was loaded into the column at a low flow rate, washed with ddH_2_O until pH ∼7.0, eluted with 9% w/v NH_4_OH (100 ml), and dried via rotary evaporation to harvest the final product (*S*)-2a. ^1^H NMR (400 MHz, D_2_O) δ 3.61 (dd, *J* = 11.8, 4.1 Hz, 1H), 3.51–3.33 (m 1H), 3.06–2.82 (m, 1H), 1.57–1.33 (m, 2H), and 0.98–0.81 (m, 3H).

### Model Generation and Substrate Docking

The structure of the mutant wh84 (K68S/N261L/I111F/V294C/E114V) was generated by PyMol program (http://www.pymol.org) using the crystallographic structure of *Sp*LeuDH (PDB ID: 3VPX, Zhao et al., 2012) as a template. The initial structure of wh84 was relaxed/repacked before docking using Rosetta relax program (Conway et al., 2014). The generated pose with the lowest Rosetta_total_score was selected as input file for docking. The cofactor NADH, substrate 1a, and NH_4_^+^ were prepared in Schrödinger Maestro software (Schrödinger, 2015). Thereafter, NADH and 1a were stepwise docked into the active site of wh84 by using Rosetta docking program (Combs et al., 2013). Thereafter, 1a was docked to the protein–NADH complex using the same procedure as NADH, only by replacing the input files. After that, NH_4_^+^ was docked to the protein–NADH-1a complex by using AutoDock Vina (Trott and Olson, 2010). For docking NH_4_^+^, a total of 16 poses were generated by Vina, and only the pose with proper interactions with D115 and 1a was kept.

## Results and Discussion

### Knowledge-Based and Structural Guided Engineering of SpLeuDH

**FIGURE 1:**
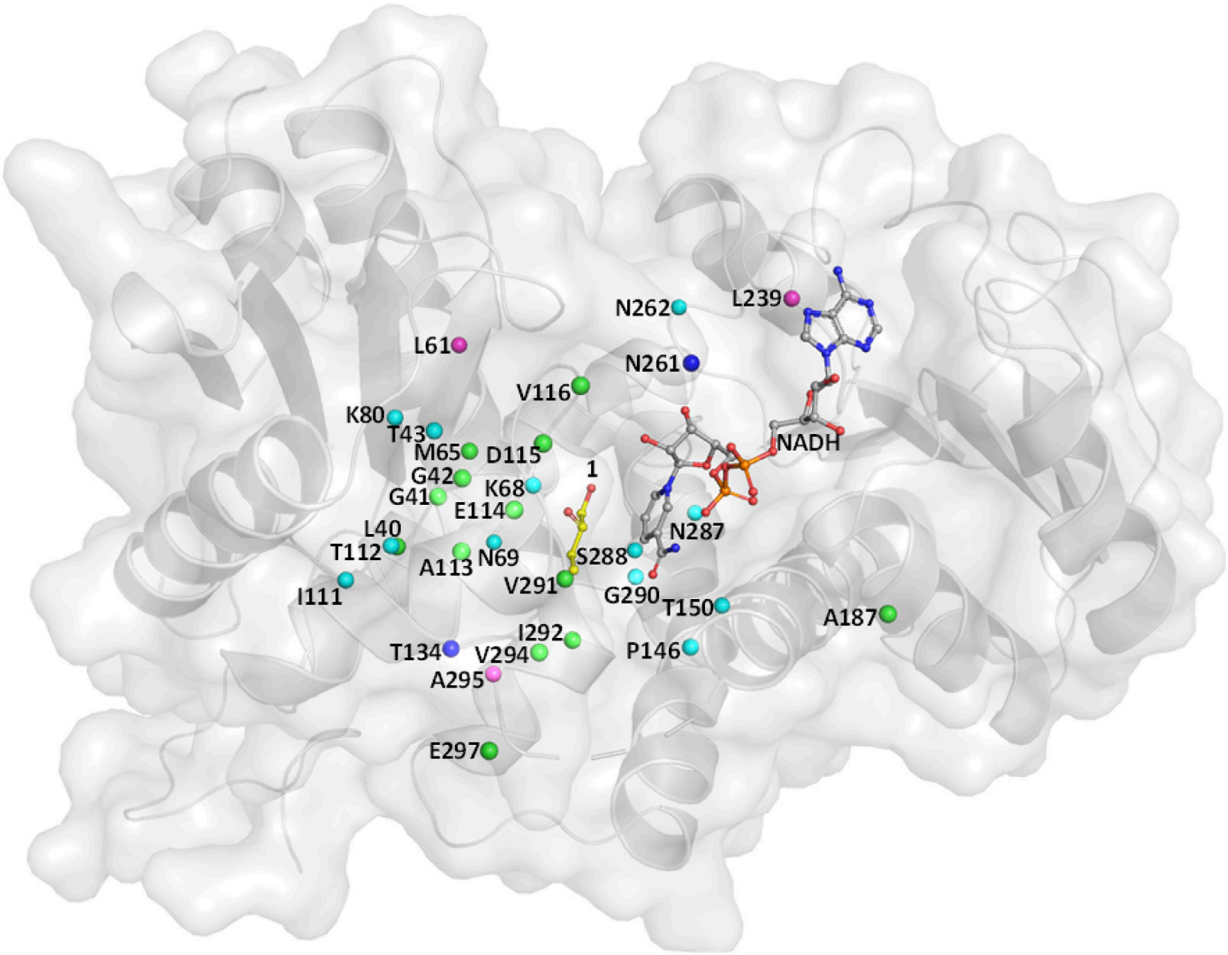
Residues lining the substrate-binding pocket of *Sp*LeuDH (PDB ID: 3VPX). The sites marked in green, cyan, and blue are obtained from the three studies Abrahamson et al. (2012); Ye et al. (2015); Chen et al. (2019), respectively. The sites marked in magenta are found in this study.

Wild-type (WT) leucine dehydrogenase from *Sporosarcina psychrophila* (*Sp*LeuDH, Accession No.: WP_067209859) has been successfully engineered to an amine dehydrogenase (*Sp*AmDH) by implanting the double mutations K68S/N261L in our recent work (Wang et al., 2020). Based on the crystallographic structure of *Sp*LeuDH (PDB ID: 3VPX, Zhao et al., 2012), this *Sp*AmDH variant (K68S/N261L, dubbed wh18) showed excellent stereoselectivity (>99% ee) in the transformation of 1a to 1b, while the catalytic efficiency was modest when it was applied in large-scale production (Wang et al., 2020). Thus, the CAST/ISM strategy was employed with the aim of enhancing the activity of *Sp*AmDH. To identify the hot spot positions that may manipulate the activity, the beneficial mutations reported in other engineering studies toward AmDHs were collected (Table 1). We anticipated that targeting these residues at the equivalent positions in *Sp*AmDH may give rise to more proficient variants. Moreover, three additional positions L61, L239, and A295 were also selected as they are situated at the substrate binding pocket (Table 1; Figure 1).

**TABLE 1:** Key residues selected for engineering *Sp*AmDH based on published data.

Entry	*Sp*AmDH site	Data collection			References
		Beneficial position	Enzyme	Comment	
1	L40	L40	*Bs*LeuDH	Lining the binding pocket	Abrahamson et al. (2012)
2	G41	G41	*Bs*LeuDH	Lining the binding pocket	Abrahamson et al. (2012)
3	G42	G42	*Bs*LeuDH	Lining the binding pocket	Abrahamson et al. (2012)
4	T43	T41	*Rs*PheDH	Within 6Å from the substrate	Ye et al. (2015)
5	M65	M65	*Bs*LeuDH	Lining the binding pocket	Abrahamson et al. (2012)
6	K68	K66	*Rs*PheDH	Carboxylate recognition	Ye et al. (2015)
7	K68	K68	*Lf*LeuDH	Altering the substrate specificity	Chen et al. (2019)
8	N69	M67	*Rs*PheDH	Within 6Å from substrate	Ye et al. (2015)
9	I111	W114	*Rs*PheDH	Within 6Å from substrate	Ye et al. (2015)
10	T112	T115	*Rs*PheDH	Within 6Å from substrate	Ye et al. (2015)
11	A113	A113	*Bs*LeuDH	Altering the substrate specificity	Abrahamson et al. (2012)
12	A113	A113	*Lf*LeuDH	Enlarging the active-site	Chen et al. (2019)
13	E114	E114	*Bs*LeuDH	Altering the substrate specificity	Abrahamson et al. (2012)
14	D115	D115	*Bs*LeuDH	Essential to the catalytic mechanism	Abrahamson et al. (2012)
15	V116	V116	*Bs*LeuDH	Altering the substrate specificity	Abrahamson et al. (2012)
16	T134	T134	*Lf*LeuDH	Enlarging the active-site	Chen et al. (2019)
17	P146	S149	*Rs*PheDH	Within 6Å from substrate	Ye et al. (2015)
18	T150	T153	*Rs*PheDH	Within 6Å from substrate	Ye et al. (2015)
19	A187	A187	*Bs*LeuDH	Altering the substrate specificity	Abrahamson et al. (2012)
20	N261	N261	*Lf*LeuDH	Responsible for altering the substrate specificity	Chen et al. (2019)
21	N262	N262	*Rs*PheDH	Interact with the carboxyl group of the natural substrate	Ye et al. (2015)
22	N287	N288	*Rs*PheDH	Within 6Å from substrate	Ye et al. (2015)
23	S288	A289	*Rs*PheDH	Within 6Å from substrate	Ye et al. (2015)
24	G290	G291	*Rs*PheDH	Within 6Å from substrate	Ye et al. (2015)
25	V291	V291	*Bs*LeuDH	Lining the binding pocket	Abrahamson et al. (2012)
26	I292	I292	*Bs*LeuDH	Altering the substrate specificity	Abrahamson et al. (2012)
27	V294	V294	*Bs*LeuDH	Lining the binding pocket	Abrahamson et al. (2012)
28	E297	E297	*Bs*LeuDH	Changing the substrate specificity	Abrahamson et al. (2012)
29	L61	—	—	Lining the binding pocket	This study
30	L239	—	—	Lining the binding pocket	This study
31	A295	—	—	Lining the binding pocket	This study

**FIGURE 2:**
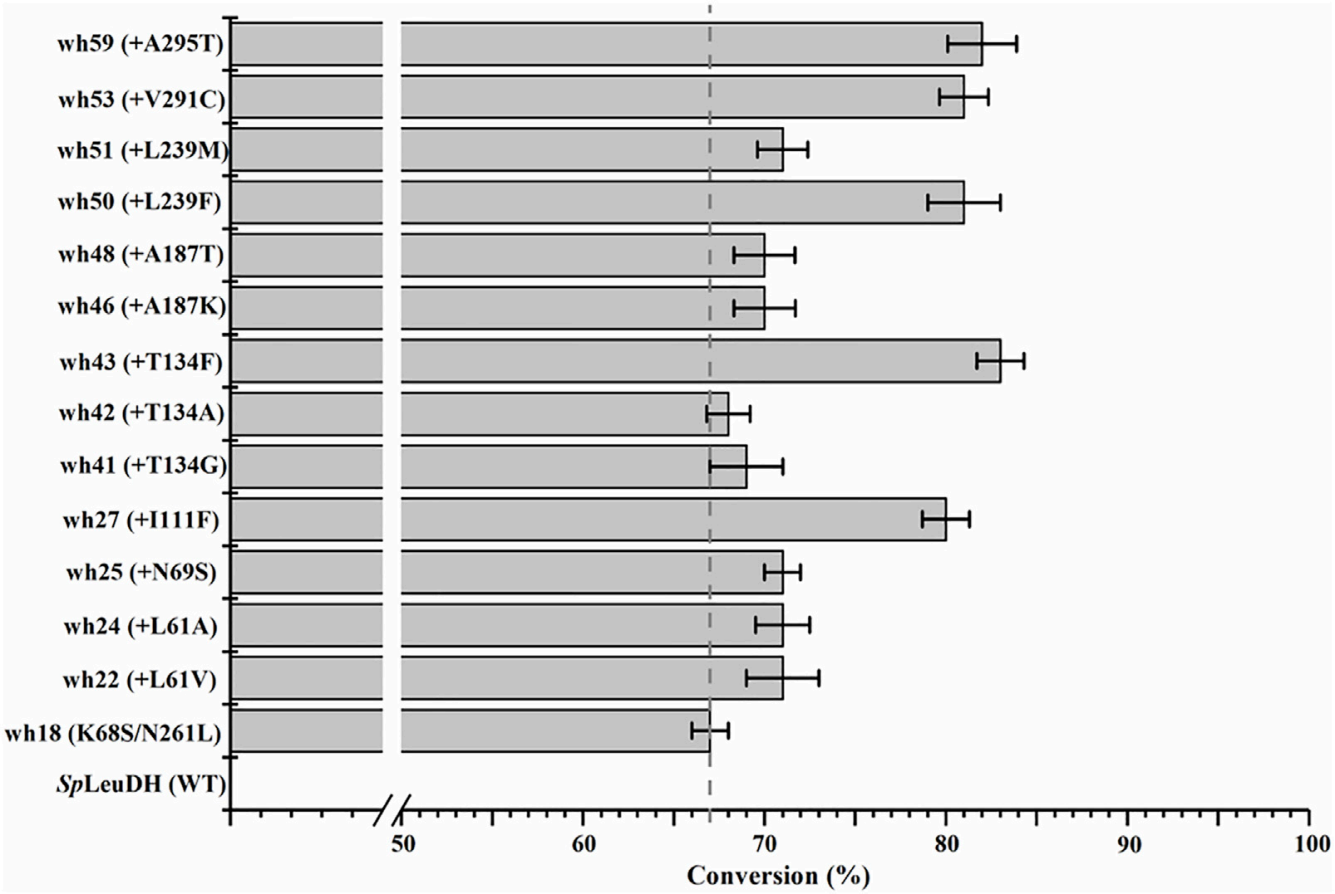
Improved variants obtained by screening the single point saturation mutagenesis libraries on the 27 positions. Reductive amination reactions were performed in a reaction mixture (0.5 ml) containing 1 M NH_4_Cl/NH_3_·H_2_O buffer (pH 8.5), 1 mM NAD^+^, 100 mM glucose, 2 mg/ml GDH cell-free extract (CFE), 6 U/ml DNase I, 20 mM 1a, and 20 mg/ml mutant CFE at 30°C, 1,000 rpm for 24 h. The conversion was detected by HPLC at least three times.

The 27 positions were then performed site-directed saturation mutagenesis using wh18 as the template, each site was substituted by the other 19 amino acids with NNK codon degeneracy (32 codons). To satisfy the 95% library coverage, 96 clones were screened for each position by using *para*-methoxy-2-amino benzamidoxime (PMA) as colorimetric probe (Mei et al., 2020). After the initial assaying, the improved variants (hits) were then picked up for further evaluation using the reaction conditions shown in Scheme 1. As a result, several active variants showing pronounced degrees of conversion for substrate 1a were obtained (Figure 2). Five of them including wh27, wh43, wh50, wh53, and wh59 showed more than 10% increment on the formation of (*S*)-1b compared to the starting enzyme wh18 (Figure 2).

### Combinatorial Engineering of SpAmDH Using Reduced Amino Acid Alphabets

After purification, the five variants were then assayed for the specific activity, three of which including wh27, wh50, and wh53 displayed a higher specific activity than that of wh18 (Table 2). In order to investigate the potential epistatic interactions operating between the individual point mutations shown in Table 2, ten additional double mutants (wh61–wh70, Supplementary Table S4) were constructed and tested using the whole-cell lysate system. As a result, the variant wh43 showed the highest conversion of 85%, while none of the ten combinatorial mutants can exceed the conversion of 80%. It suggests that no additive effects among the five single mutations I111F, T134F, L239F, V291C, and A295T were in pairs, and the triple to quintuple mutants were therefore not constructed further.

**TABLE 2:** Specific activity of *Sp*AmDH mutants toward substrate 1a.

Code	Mutants	Specific activity (U/mg)^a^
wh18	K68S/N261L	0.118 ± 0.005
wh27	K68S/N261L/I111F	0.177 ± 0.03
wh43	K68S/N261L/T134F	0.07 ± 0.005
wh50	K68S/N261L/L239F	0.15 ± 0.017
wh53	K68S/N261L/V291C	0.167 ± 0.005
wh59	K68S/N261L/A295T	0.054 ± 0.007

a Reaction was performed in 1 M NH_4_Cl/NH_3_·H_2_O buffer (pH 8.5) containing 0.2 mM NADH, 1 mg/ml purified enzyme and 10 mM substrate 1a, at 30°C for 5 min.

In parallel, we noted that the three mutants with improved activity, shown in Table 2, are all composed of the substitutions of phenylalanine and cysteine (e.g., I111F, L239F, and V291C), indicating that hydrophobic interaction and steric hindrance may play key roles in tailoring the activity. Therefore, phenylalanine and cysteine were chosen as building blocks for the further saturation mutagenesis to determine the interactions among the positions in adjacent to residues 111, 239, and 291. As such, two focused libraries A and B were constructed using F and C as reduced amino acid alphabets based on the double-code saturation mutagenesis (DCSM) concept (Sun et al., 2016): library A using wh53 as template with residues M65, S68, N69, and S288 involved, while library B applying wh27 as template with residues L40, A113, T134, and V294 arrested. As a result, two improved mutants wh76 (K68S/N261L/V291C/S68C/N69C) and wh81 (K68S/N261L/I111F/V294C) were obtained from libraries A and B, respectively. The variants wh76 and wh81 gave a conversion of 87 and 90% in the transformation of 1a, respectively, with >99% ee.

### Third Round of Mutagenesis Toward SpAmDH, Characterization, and Preparative-Scale Reduction

**FIGURE 3:**
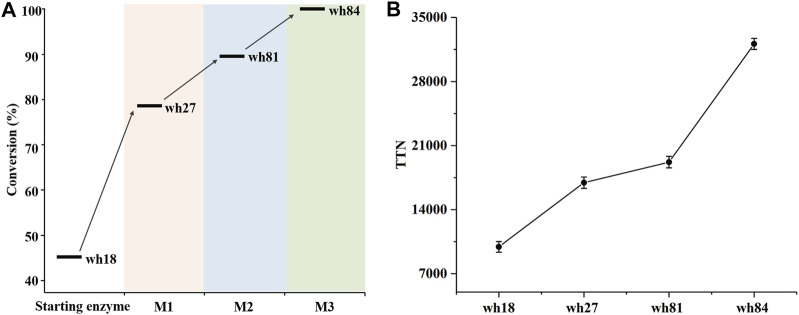
Conversion **(A)** and TTN **(B)** analysis of the key variants using 40 mM substrate 1a. M1∼M3 depicted the three rounds of mutagenesis.

The variant wh81 was further used as template for the third round of mutagenesis. In this scenario, an important single mutation E114V was considered because it has been reported that this residue can function on the ammonia activation, and thereby affecting the enzyme activity (Abrahamson et al., 2012; Chen et al., 2018; Patil et al., 2018; Lee et al., 2021). To our delight, the resultant quintuple variant wh84 (K68S/N261L/I111F/V294C/E114V) showed 99% conversion and >99% ee toward 1a. Taken together, after three rounds of mutagenesis, the conversion of 1a directed by the *Sp*AmDH mutants was successfully elevated from 46 to 99% (Supplementary Figure S3; Figure 3A). Thereafter, the best mutants in each round of mutagenesis were then purified and characterized by total turnover numbers (TTN), thermostability, and enzyme kinetics. Likewise, wh27, wh81, and wh84 increased the TTN stepwise for substrate 1a (Figure 3B). In particular, wh84 increased the TTN up to 32108, which is 3.2-fold in contrast to that of wh18.

To evaluate the enzyme robustness of the engineered variants, thermostability was assessed by measuring *T*_*50*_^*15*^, the temperature at which 50% of the enzyme activity is lost following a heat treatment for 15 min. Intriguingly, all of the three variants constructed based on wh18 showed comparable *T*_*50*_^*15*^ values, reflecting no trade-off between the thermostability and the improved activity (Table 3). Kinetic studies showed that wh84 has the highest catalytic efficiency (*k*_cat_/*K*_m_) (0.346 s^−1^mM^−1^) among all the mutants, a more than 3.9-fold increase to the starting template wh18.

**TABLE 3:** Thermostability and kinetic parameters of purified *Sp*AmDH mutants for substrate 1a.

Code	Thermostabiliy *T*_*50*_^*15*^ (°C)^a^	Mutations	*K*_m_ (mM)^b^	*k*_cat_ (s^−1^)^b^	*k*_cat_/*K*_m (_s^−1^mM^−1^_)_ ^b^	Specific activity (U/mg)^b^
wh18	51	K68S/N261L	9.45 ± 1.44	0.83 ± 0.05	0.088	0.118 ± 0.005
wh27	50	K68S/N261L/I111F	18.52 ± 1.42	1.74 ± 0.07	0.094	0.177 ± 0.030
wh81	51	K68S/N261L/I111F/V294C	21.5 ± 3.37	3.14 ± 0.27	0.146	0.362 ± 0.019
wh84	53	K68S/N261L/I111F/E114V/V294C	17.53 ± 1.11	6.07 ± 0.19	0.346	0.884 ± 0.044

a For determining thermostability, purified *Sp*AmDH enzymes (1 mg/ml) were incubated at different temperatures (30–70°C) for 15 min, followed by measuring the residual activity toward substrate 1a.

b For assaying kinetic parameters, the reaction was performed in 1 M NH_4_Cl/NH_3_·H_2_O buffer (pH 8.5) containing 0.2 mM NADH and 0.5–30 mM substrate 1a, at optimum temperatures (50°C) for 2 min. Michaelis–Menten plots are shown in Supplementary Figure S2.

**FIGURE 4:**
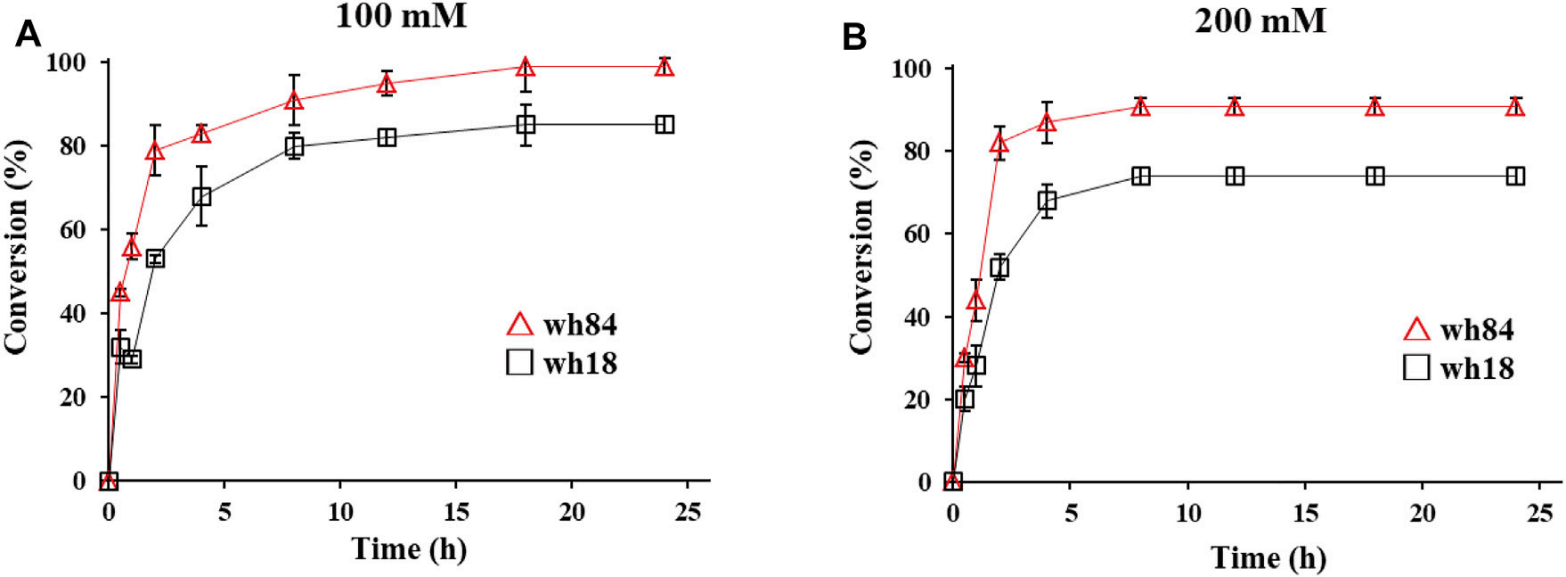
Preparative-scale of the asymmetric amination reactions of substrate 1a 100 mM **(A)** and 200 mM **(B)** catalyzed by wh18 and wh84 (□: wh18, △: wh84). Reaction system: 0.1 g/ml wet cell, 1 M NH_4_Cl/NH_3_·H_2_O buffer (pH 8.5), 1 mM NAD^+^, 100 mM glucose, 2 mg/ml GDH CFE, 6 U/mL DNase I, 1 mg/ml lysozyme, and 1a (100 and 200 mM) were mixed in Erlenmeyer flask. The reaction was performed at 30°C, 220 rpm for 24 h. The samples taken at 0, 1, 2, 3, 4, 8, 12, 18, and 24 h were prepared and analyzed by HPLC.

Preparative-scale amination reactions were performed using whole-cell lysates as catalysts for the selected variants in 10 ml of reaction volume, with 100 mM substrate 1a. The mutant wh84 achieved an excellent conversion (>99%), with the high enantioselectivity of >99% ee within 18 h, while wh18 used as a control only showed ca. 80% conversion (Figure 4A). (*S*)-2b was subsequently isolated as pure form (164 mg, 62% yield) in the reaction system catalyzed by wh84. To further examine the catalytic potential of wh84, the concentration of 1a was raised up to 200 mM. Interestingly, wh84 enabled 91% conversion (Figure 4B).

### Gaining Insight on the Improved Activity of SpAmDH

**FIGURE 5:**
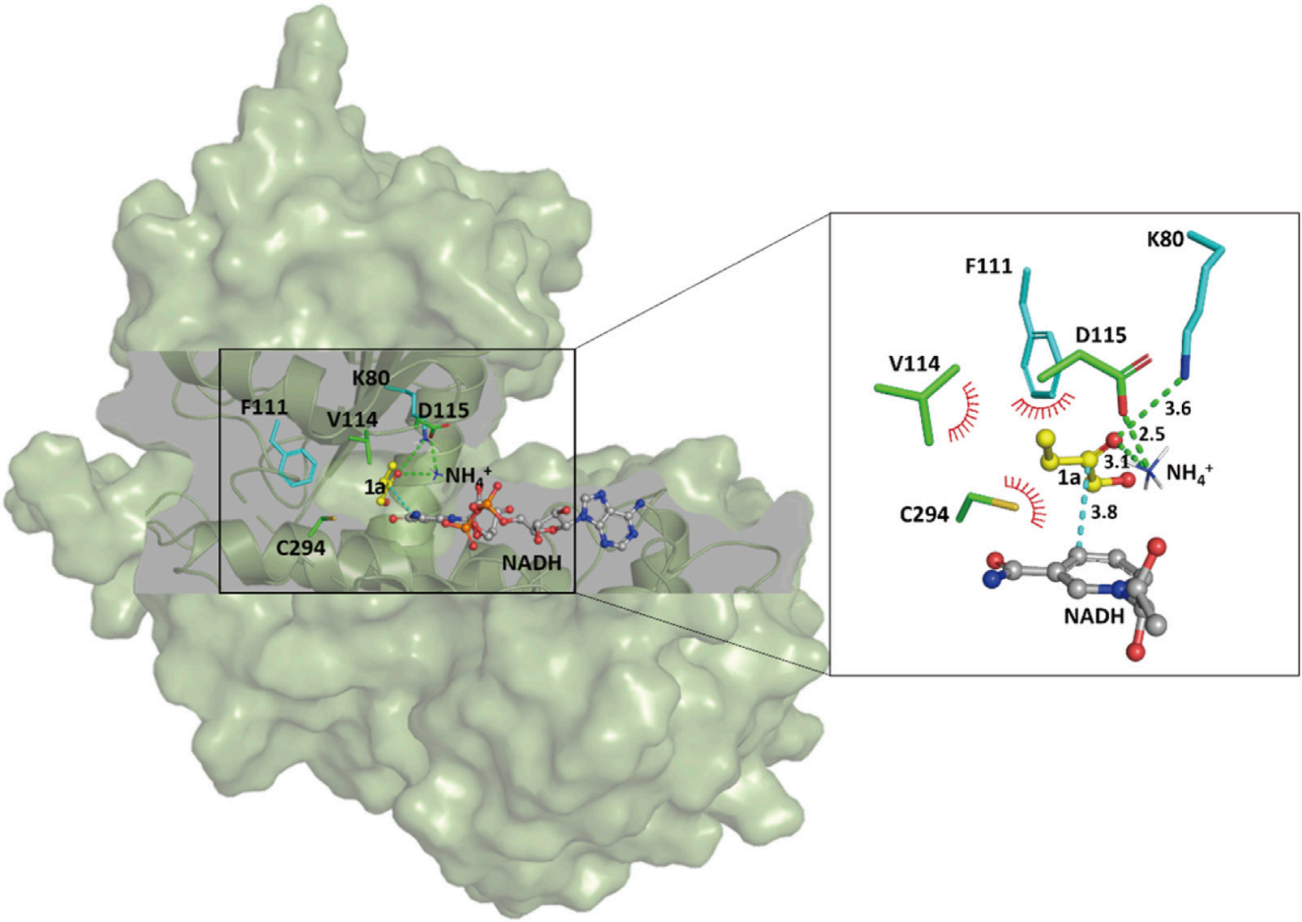
Docking analysis of substrate 1a in the catalytic pocket of the *Sp*AmDH variant wh84. Green lines and red spikes represent the hydrogen bonds and the penitential hydrophobic interactions, respectively. The cyan line indicates the nucleophilic attack. Distances are shown in angstroms.

In order to shed light on the improved activity of the *Sp*AmDH variant wh84, computational docking analyses were performed to gain insights into the relationship between reshaping of the active site and the effect on activity. The homology structure of wh84 was directly constructed based on the X-ray structure of *Sp*LeuDH (PDB ID: 3VPX, Zhao et al., 2012) by introducing the corresponding mutations in PyMol program (http://www.pymol.org). After relaxing the initial structure of wh84, cofactor NADH, substrate 1a, and ammonium ion (NH_4_^+^) were docked into the active site one after another to generate the protein–NADH-1a–NH_4_^+^ quaternary complex. It is of interest to note that the catalytic sites K80 and D115 form hydrogen bond interactions with the substrate carbonyl group and the ammonium ion, while the substitutions I111F, E114V, and V294C contribute to hydrophobic interactions with the substrate carbon chain (Figure 5). These strengthened interactions may benefit the substrate recognition and orientation, thereby promoting the activity and maintaining the stereoselectivity. Moreover, on the basis of the proposed mechanism of the engineered AmDHs and their parent AADHs (Sharma et al., 2017), this preferred binding orientation of 1a with respect to NADH determines the *Re* face of the C=O bond undergoes nucleophilic attack and profits the (*S*)-configure product, which is consistent with the experimental results.

### Substrate Scope Analysis Toward Other Prochiral Hydroxy ketones

**FIGURE 6:**
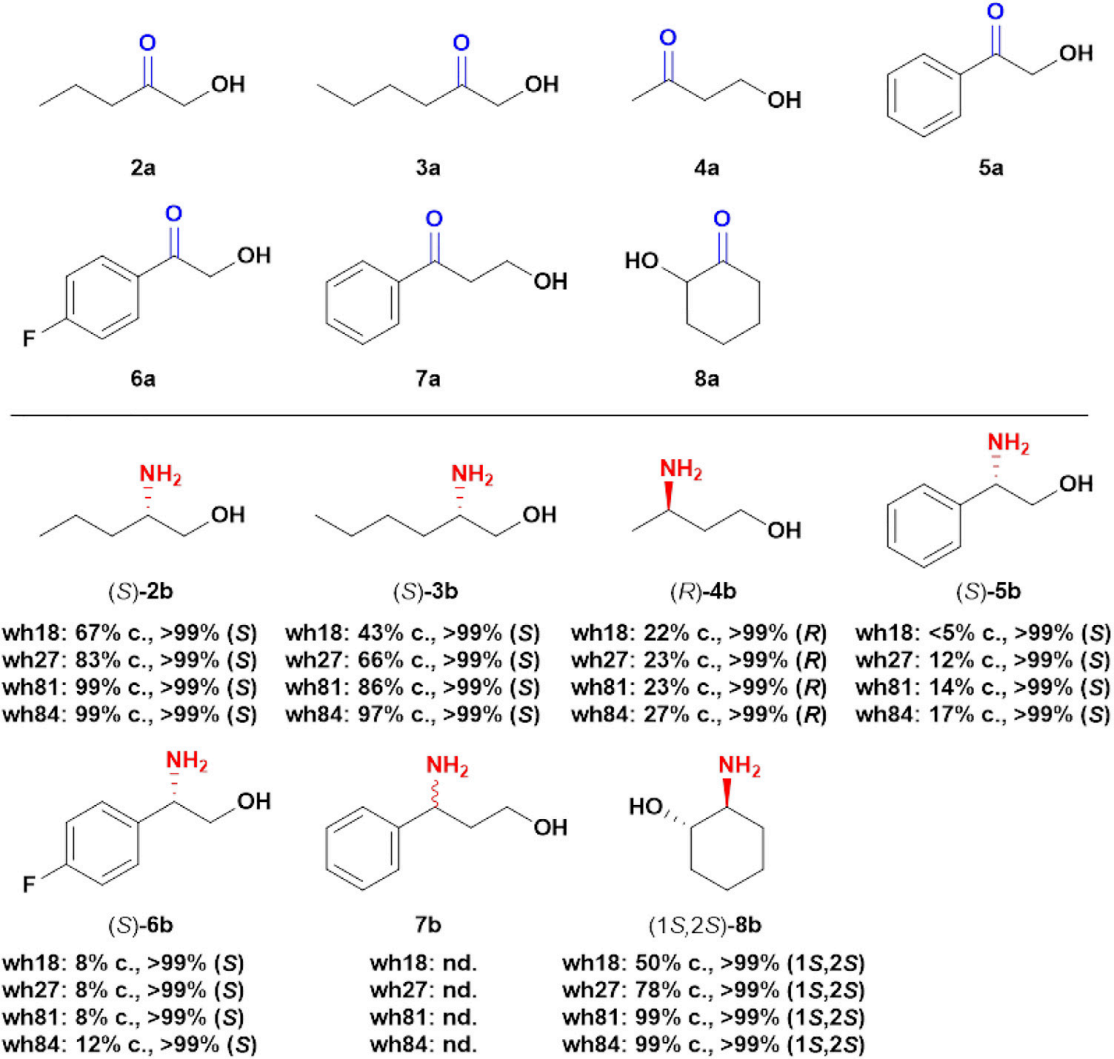
Asymmetric transformations of hydroxy ketones. Reaction system: 0.1 g/ml wet cell, 1 M NH_4_Cl/NH_3_·H_2_O buffer (pH 8.5), 1 mM NAD^+^, 100 mM glucose, 2 mg/ml GDH CFE, 6 U/ml DNase I, 1 mg/ml lysozyme, and 2a-8a (5 mM) were mixed in 2 ml Eppendorf tubes. The reaction was performed at 30°C, 1,000 rpm for 24 h in a thermostatic metal bath. c, conversion; nd, not detectable.

With the aim to explore the catalytic potential of the engineered variants toward other prochiral hydroxy ketones, seven structurally different substrates were assayed using wh18, wh27, wh81, and wh84 as catalysts (Figure 6). For the four aliphatic compounds 2a–4a and cyclic ketone 8a, the variants showed high stereoselectivity (>99%), and interestingly, the conversions were elevated from wh18 to wh84 in all the four cases (Supplementary Figures S4–S6, S10). For example, the conversion of 3a was improved from 43% (wh18) to 97% (wh84). However, in the scenario of the aromatic substrates, the four variants displayed relatively lower activity in the asymmetric reduction of 5a–7a (Figure 6), while the stereoselectivities were still inherently maintained (>99%, Supplementary Figures S7–S9).

## Conclusion

Reductive amination of carbonyl compounds employing AmDHs is an attractive route for the biosynthesis of chiral amino alcohols. It provides several advantages, including lower costs (the amino donor (NH_4_^+^) is inexpensive, and water is the main byproduct), elimination of heavy metals, and high stereoselectivity. As such, AmDHs have been reported in the selective synthesis of chiral compounds used as pharmaceutical intermediates. However, the limited catalytic efficiency can be a major obstacle to its industrial application. This work reports the engineering of *Sp*AmDH with the aim to improve the activity. After three rounds of CAST/ISM-guided mutagenesis, mutant wh84 was obtained with the best performance toward substrate 1a, resulting in an excellent TTN (32108) and a *k*_cat_/*K*_m_ value of 0.346 s^−1^ mM^−1^, amounting to 3.2-fold and 3.9-fold improvements relative to the starting enzyme, respectively, while maintaining the high enantioselectivity (>99% *ee*). In the 100 mM preparative reaction, the conversion of 1a catalyzed by wh84 was up to 99% with a 62% yield, which is comparable with the recent work that employed an engineered AmDH from *Lysinibacillus fusiformis* as catalyst (Chen et al., 2019).

Overall, this work paves the way toward engineering AmDHs with increased activity and also expands the biocatalytic toolbox for asymmetric reductive aminations, and should prove useful insights for further development of other AmDHs as catalysts in the biosynthesis of enantiopure amino alcohols.

## Data Availability

The original contributions presented in the study are included in the article/Supplementary Material, and further inquiries can be directed to the corresponding authors.
