# Supplementary material for: Biosynthesis of Chiral Amino Alcohols via an Engineered Amine Dehydrogenase in E. coli
Source: Front Bioeng Biotechnol. 2022 Jan 5;9:778584. doi: 10.3389/fbioe.2021.778584 (PMC8766677; doi:10.3389/fbioe.2021.778584)

Supplementary Material

Content

**Scheme**

Scheme S1. Chiral α-amino alcohols as building blocks (in red color) in the representative pharmaceuticals. 2

**Tables**

Table S1. Primers for construction of single-point saturation mutation library 3

Table S2. Primers used in combined saturation mutant libraries 5

Table S3. Analysis conditions of HPLC ^a^ 6

Table S4. Conversions of **1a** catalyzed by combinatorial mutants 8

**Figures**

Figure S1. SDS-PAGE analysis of purified *Sp*AmDH variants. 9

Figure S2. Michaelis-Menten curve of NADH to NAD^+^ for **1a** catalyzed by *Sp*AmDH variants: wh18 (A), wh27 (B), wh81 (C) and wh84 (D). 10

Figure S3. HPLC spectra of (*S*)-**1b** synthesized from **1a** (40 mM) by *Sp*AmDH variants. 11

Figure S4. HPLC spectra of (*S*)-**2b** synthesized from **2a** (5 mM) by *Sp*AmDH variants. 12

Figure S5. HPLC spectra of (*S*)-**3b** synthesized from **3a** (5 mM) by *Sp*AmDH variants. 13

Figure S6. HPLC spectra of (*R*)-**4b** synthesized from **4a** (5 mM) by *Sp*AmDH variants. 14

Figure S7. HPLC spectra of (*S*)-**5b** synthesized from **5a** (5 mM) by *Sp*AmDH variants. 15

Figure S8. HPLC spectra of (*S*)-**6b** synthesized from **6a** (5 mM) by *Sp*AmDH variants. 16

Figure S9. HPLC spectra of **7b** synthesized from **7a** (5 mM) by *Sp*AmDH variants. 17

Figure S10. HPLC spectra of (1*S*, 2*S*)-**8b** synthesized from **8a** (5 mM) by *Sp*AmDH variants. 18

**NMR spectra**

(*S*)-2-aminobutan-1-ol (**1b**) 19


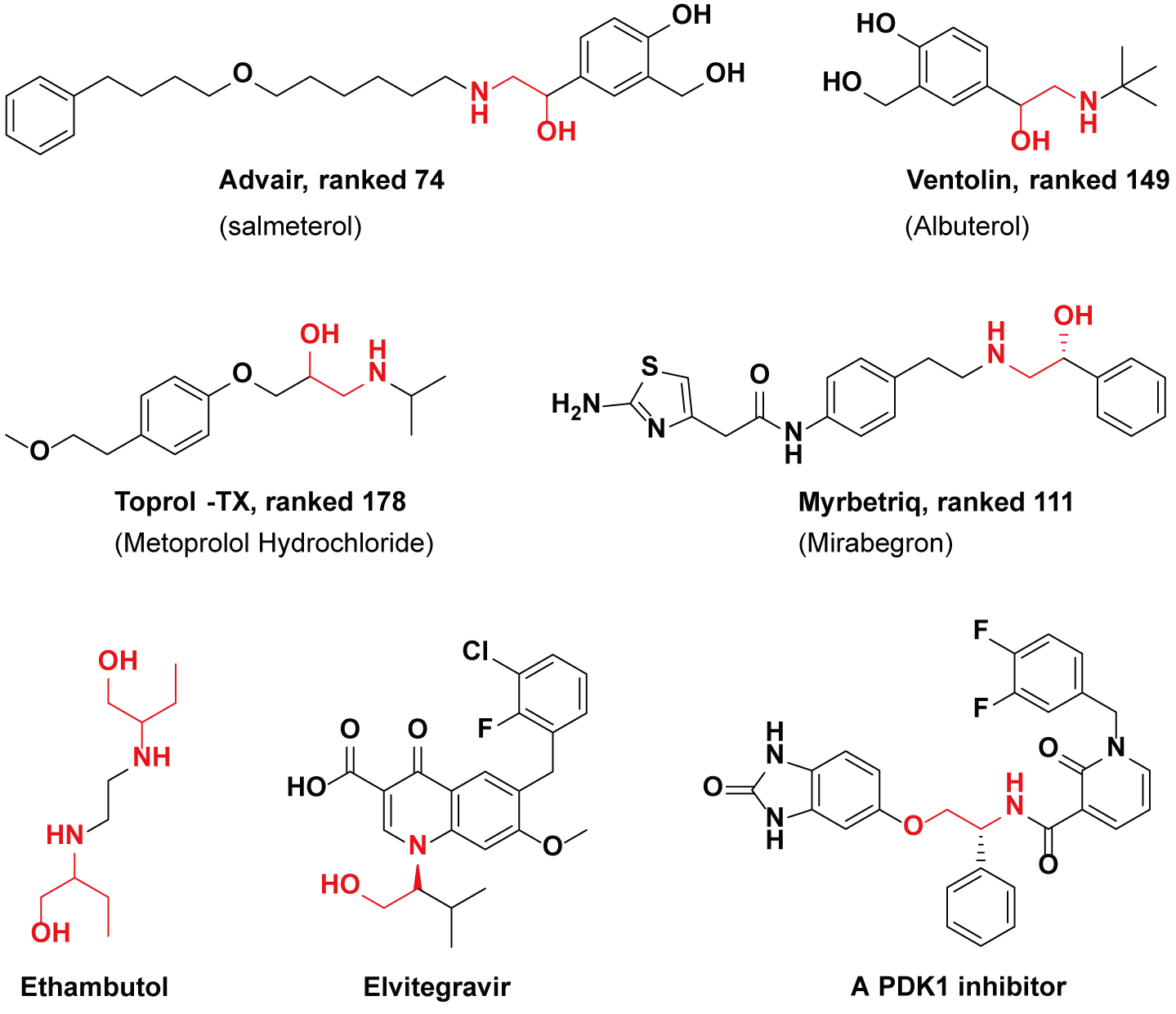


Scheme S1. Chiral α-amino alcohols as building blocks (in red color) in the representative pharmaceuticals.

Table S1. Primers for construction of single-point saturation mutation library

| **Site** | **Primers** |
| --- | --- |
| L40-F | ACCCTGGGCCCGGCC**NDT/VMA/ATG/TGG**GGTGGTACACGTATG |
| L40-R | CAGATCCATATCTGCTTCGGTGGTGCCAACATC |
| G41-F | CTGGGCCCGGCCCTG**NDT/VMA/ATG/TGG**GGTACACGTATGTGG |
| G41-R | CAGATCCATATCTGCTTCGGTGGTGCCAACATC |
| G42-F | GGCCCGGCCCTGGGT**NDT/VMA/ATG/TGG**ACACGTATGTGGACC |
| G42-R | CAGATCCATATCTGCTTCGGTGGTGCCAACATC |
| T43-F | CCGGCCCTGGGTGGT**NDT/VMA/ATG/TGG**CGTATGTGGACCTAT |
| T43-R | CAGATCCATATCTGCTTCGGTGGTGCCAACATC |
| L61-F | GAAGATGCACTGCGC**NDT/VMA/ATG/TGG**GCACGTGGTATGACC |
| L61-R | AACGCCCTGAACGGCAACGGTTTTACCTGCCAG |
| M65-F | CGCCTGGCACGTGGT**NDT/VMA/ATG/TGG**ACCTATAGTAATGCA |
| M65-R | AACGCCCTGAACGGCAACGGTTTTACCTGCCAG |
| N69-F | GGTATGACCTATAGT**NDT/VMA/ATG/TGG**GCAGCAGCAGGCCTG |
| N69-R | AACGCCCTGAACGGCAACGGTTTTACCTGCCAG |
| I111-F | CTGAATGGTCGTTAT**NDT/VMA/ATG/TGG**ACCGCAGAAGATGTT |
| I111-R | AACGCCCTGAACGGCAACGGTTTTACCTGCCAG |
| T112-F | AATGGTCGTTATATT**NDT/VMA/ATG/TGG**GCAGAAGATGTTGGC |
| T112-R | AACGCCCTGAACGGCAACGGTTTTACCTGCCAG |
| A113-F | GGTCGTTATATTACC**NDT/VMA/ATG/TGG**GAAGATGTTGGCACC |
| A113-R | AACGCCCTGAACGGCAACGGTTTTACCTGCCAG |
| E114-F | CGTTATATTACCGCA**NDT/VMA/ATG/TGG**GATGTTGGCACCACC |
| E114-R | AACGCCCTGAACGGCAACGGTTTTACCTGCCAG |
| D115-F | GTTATATTACCGCAGAA**NDT/VMA/ATG/TGG**GTTGGCACCACCG |
| D115-R | AACGCCCTGAACGGCAACGGTTTTACCTGCCAG |
| V116-F | ATTACCGCAGAAGAT**NDT/VMA/ATG/TGG**GGCACCACCGAAGCA |
| V116-R | AACGCCCTGAACGGCAACGGTTTTACCTGCCAG |
| T134-F | GAAACCGATTATGTT**NDT/VMA/ATG/TGG**GGCACCAGCGCAGGT |
| T134-R | TTCATTAATACCAACTGCGGTGGCGCCAAAAGC |
| P146-F | GGTAGTAGTGGCAAT**NDT/VMA/ATG/TGG**AGCCCGGTGACCGCA |
| P146-R | TTCATTAATACCAACTGCGGTGGCGCCAAAAGC |
| T150-F | AATCCGAGCCCGGTG**NDT/VMA/ATG/TGG**GCATACGGTATCTAT |
| T150-R | TTCATTAATACCAACTGCGGTGGCGCCAAAAGC |
| A187-F | GATGTTGGCACCACCGAAGCAGATATGGATCTG |
| A187-R | CAGATATTCGCACAG**AHN/TKB/CAT/CCA**ATAGGCAACATTACC |
| L239-F | CTGGCAGGTAAAACCGTTGCCGTTCAGGGCGTT |
| L239-R | ATTAATAATTGCACC**AHN/TKB/CAT/CCA**TGCGCACGGGGCAAA |
| N262-F | CTGGCAGGTAAAACCGTTGCCGTTCAGGGCGTT |
| N262-R | GGTTTCTTTCAGCTG**AHN/TKB/CAT/CCA**CAGTGCGCTACCGGC |
| N287-F | GCTTTTGGCGCCACCGCAGTTGGTATTAATGAA |
| N287-R | AATCACACCGCCACT**AHN/TKB/CAT/CCG**AATAACATAATCCGG |
| S288-F | GCTTTTGGCGCCACCGCAGTTGGTATTAATGAA |
| S288-R | ATTAATCACACCGCC**AHN/TKB/CAT/CCA**ATTAATAACATAATC |
| G290-F | GCTTTTGGCGCCACCGCAGTTGGTATTAATGAA |
| G290-R | GGCAACATTAATCAC**AHN/TKB/CAT/CCA**GCCACTATTAATAAC |
| V291-F | GCTTTTGGCGCCACCGCAGTTGGTATTAATGAA |
| V291-R | ATCGGCAACATTAAT**AHN/TKB/CAT/CCA**ACCGCCACTATTAAT |
| I292-F | GCTTTTGGCGCCACCGCAGTTGGTATTAATGAA |
| I292-R | TTCATCGGCAACATT**AHN/TKB/CAT/CCA**CACACCGCCACTATT |
| V294-F | GCTTTTGGCGCCACCGCAGTTGGTATTAATGAA |
| V294-R | ATCCAGTTCATCGGC**AHN/TKB/CAT/CCA**ATTAATCACACCGCC |
| A295-F | GCTTTTGGCGCCACCGCAGTTGGTATTAATGAA |
| A295-R | GCCATCCAGTTCATC**AHN/TKB/CAT/CCA**AACATTAATCACACC |
| E297-F | GCTTTTGGCGCCACCGCAGTTGGTATTAATGAA |
| E297-R | ATTATAGCCATCCAG**AHN/TKB/CAT/CCA**ATCGGCAACATTAAT |

Table S2. Primers used in combined saturation mutant libraries

| **Library** | **Primer mix** | **Name** | **Sequence (5’-3’)** |
| --- | --- | --- | --- |
| A  (F-C) | F1 | AmDH-M65TKT-S68TBT-N69TKT-F  AmDH-M65TKT-S68TBT-N69AAT-F  AmDH-M65ATG-S68TBT-N69TKT-F  AmDH-M65ATG-S68TBT-N69AAT-F | CGCCTGGCACGTGGT**TKT**ACCTAT**TBTTKT**GCAGCAGCAGGCCTG  CGCCTGGCACGTGGT**TKT**ACCTAT**TBTAAT**GCAGCAGCAGGCCTG  CGCCTGGCACGTGGT**ATG**ACCTAT**TBTTKT**GCAGCAGCAGGCCTG  CGCCTGGCACGTGGT**ATG**ACCTAT**TBTAAT**GCAGCAGCAGGCCTG |
|  | R1 | AmDH-S288AVA-R | ATCGGCAACATTAATACAACCGCC**AVA**ATTAATAACATAATC |
| B  (F-C) | F2 | AmDH-L40TKT-F  AmDH-L40WT-F | ACCCTGGGCCCGGCC**TKT**GGTGGTACACGTATG  ACCCTGGGCCCGGCC**CTG**GGTGGTACACGTATG |
|  | R2 | AmDH-A113AMA-R  AmDH-A113WT-R | CAGATCCATATCTGCTTCGGTGGTGCCAACATCTTC**AMA**GGTAAAATAACGACCATTCAG  CAGATCCATATCTGCTTCGGTGGTGCCAACATCTTC**CGC**GGTAAAATAACGACCATTCAG |
|  | F3 | AmDH-T134TKT-F  AmDH-T134WT-F | CCGAAGCAGATATGGATCTGATTAATCTGGAAACCGATTATGTT**TKT**GGCACCAGCGCAGGT  CCGAAGCAGATATGGATCTGATTAATCTGGAAACCGATTATGTT**ACC**GGCACCAGCGCAGGT |
|  | R3 | AmDH-V294AMA-R  E162-V294WT-R | ATCCAGTTCATCGGC**AMA**ATTAATCACACCGCC  ATCCAGTTCATCGGC**CAC**ATTAATCACACCGCC |

Table S3. Analysis conditions of HPLC ^a^

| **Substrates** | **Products** | **Retention time (min)** | **Products** | **Retention time (min)** |
| --- | --- | --- | --- | --- |
| **1a** |  | 12.59 |  | 17.88 |
| **2a** |  | 15.66 |  | 17.23 |
| **3a** |  | 18.68 |  | 19.54 |
| **4a** |  | 13.16 |  | 16.58 |
| **5a** |  | 14.68 |  | 16.10 |
| **6a** |  | 15.28 |  | 16.83 |
| **7a** |  | 17.97 |  | 17.01 |
| **8a** |  | 13.62 |  | 17.98 |

^a^ The reaction mixture was mixed with 100 μL sample, 30 μL of Marfey’s reagent (14 mM), 80 μL NaHCO_3_ (1 M) and 200 μL DMSO at 80 ℃, 1,000 rpm for 10 min. Finally, 10 μL HCl (4 M) was added to stop the reaction. HPLC conditions: Zorbax SB-C18 column (4.6×150 mm, 5 μm), detection wavelength: 340 nm, temperature: 25 °C, flow rate: 1 mL/min, loading volume: 10 μL, mobile phase buffer A: ddH_2_O (0.1% trifluoroacetic acid), buffer B: methanol (0.1% trifluoroacetic acid), gradient program: 40% B; hold for 6 min; increase B to 60% in 9 min, hold for 3 min; decrease B to 40% in 2 min, hold for 5 min.

Table S4. Conversions of 1a catalyzed by combinatorial mutants

| **Code** | **Mutations** | **Conversion (%)^a^** |
| --- | --- | --- |
| wh18 | K68S/N261L | 67 |
| wh27 | K68S/N261L/I111F | 80 |
| wh43 | K68S/N261L/T134F | 85 |
| wh50 | K68S/N261L/L239F | 81 |
| wh53 | K68S/N261L/V291C | 81 |
| wh59 | K68S/N261L/A295T | 82 |
| wh61 | K68S/N261L/I111F/T134F | 80 |
| wh62 | K68S/N261L/I111F/L239F | 66 |
| wh63 | K68S/N261L/I111F/V291C | 73 |
| wh64 | K68S/N261L/I111F/A295T | 44 |
| wh65 | K68S/N261L/T134F/L239F | 78 |
| wh66 | K68S/N261L/T134F/V291C | 50 |
| wh67 | K68S/N261L/T134F/A295T | 60 |
| wh68 | K68S/N261L/L239F/V291C | 62 |
| wh69 | K68S/N261L/L239F/A295T | 43 |
| wh70 | K68S/N261L/V291C/A295T | 43 |

**^a^** Reaction system: 0.1 g/mL wet cell of mutant, 1 mg/mL Lysozyme, 6 U/mL DNase I, 1 M NH_4_Cl/NH_3_·H_2_O buffer (pH 8.5), 1 mM NAD^+^, 100  mM Glucose, 2 mg/mL GDH cell free extract (CFE), 6 U/mL DNase I and 20 mM **1a** at 30 °C, 1000 rpm for 24 h. The conversion was detected by HPLC.


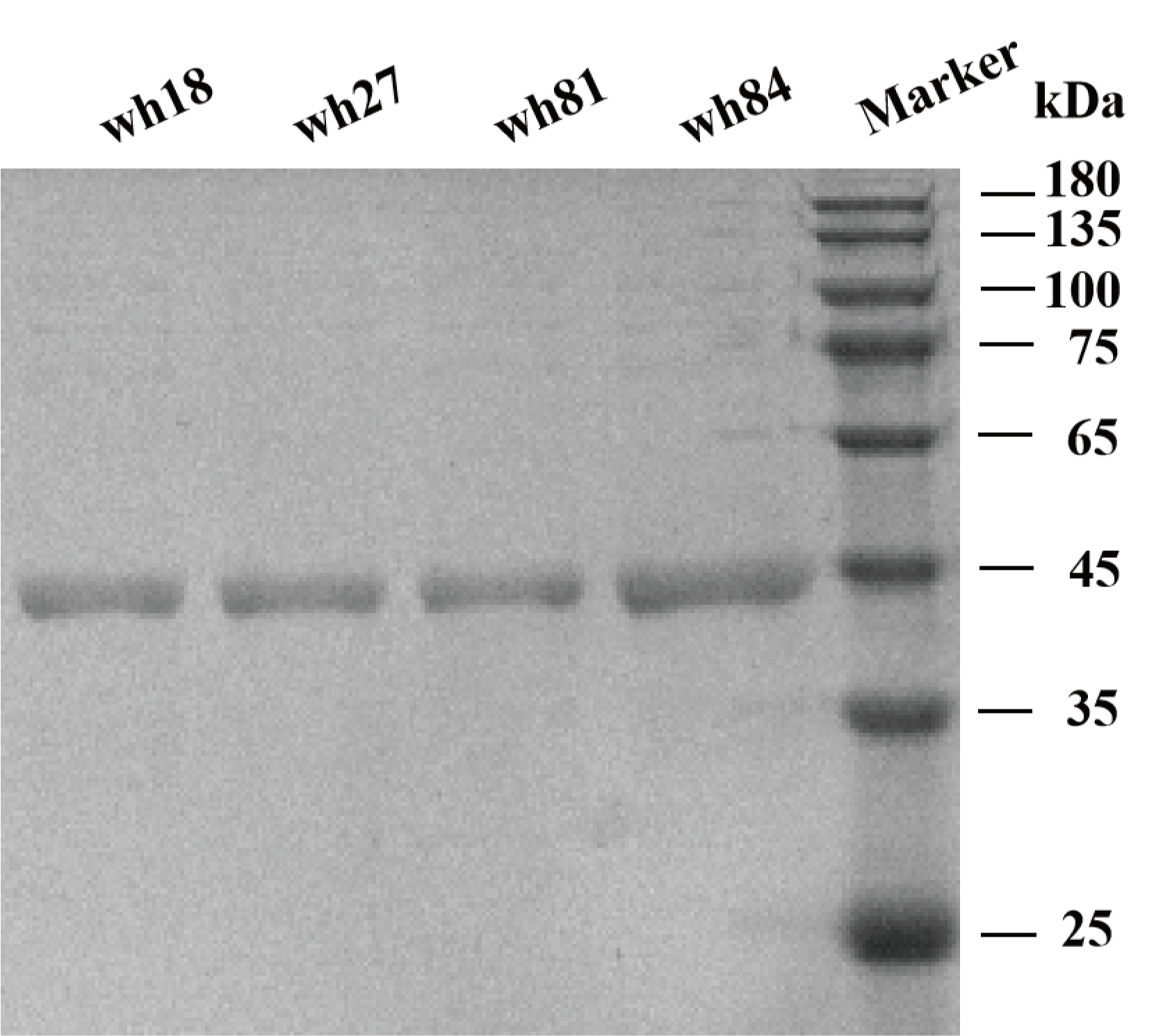


Figure S1. SDS-PAGE analysis of purified *Sp*AmDH variants.


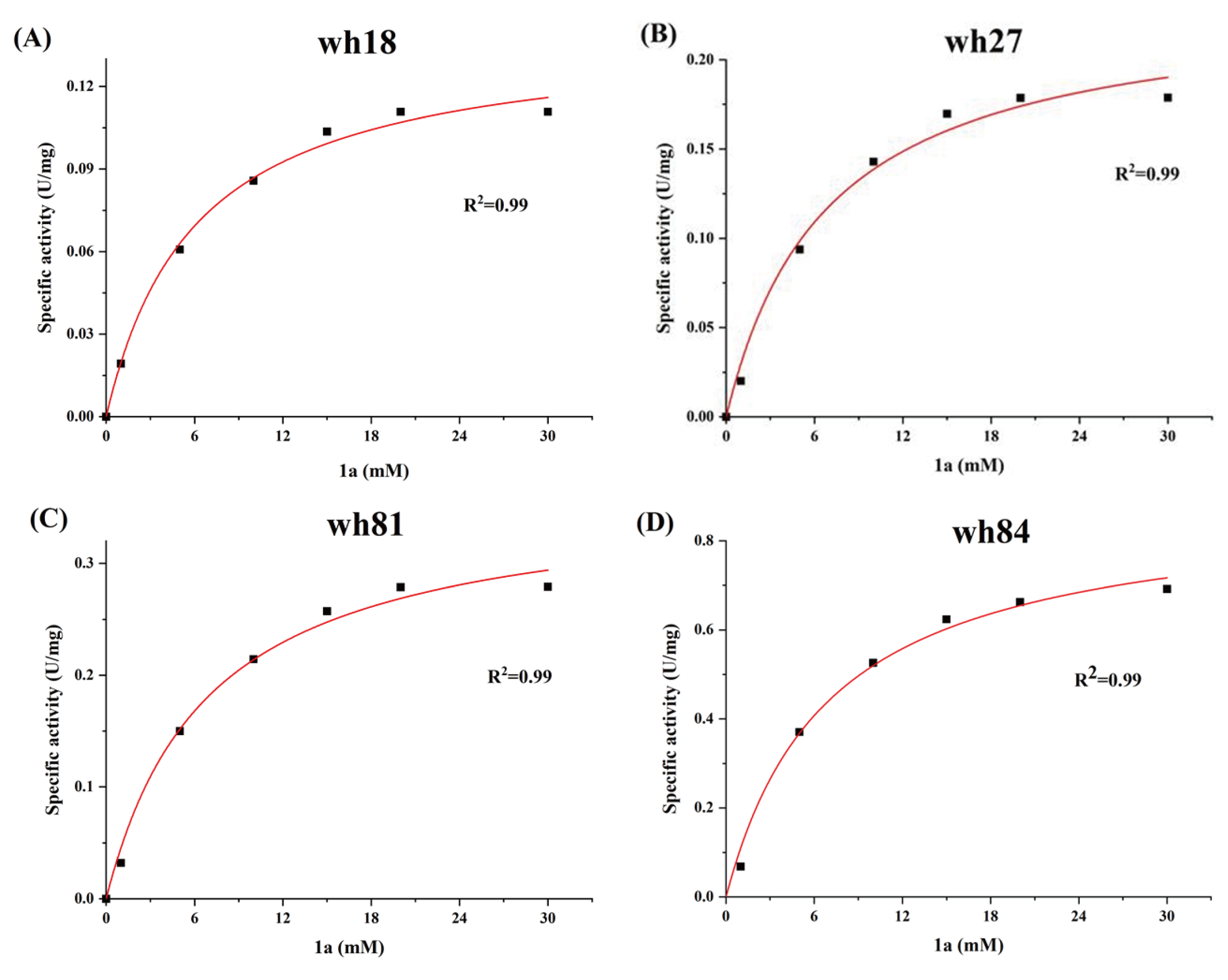


Figure S2. Michaelis-Menten curve of NADH to NAD^+^ for 1a catalyzed by *Sp*AmDH variants: wh18 (A), wh27 (B), wh81 (C) and wh84 (D).


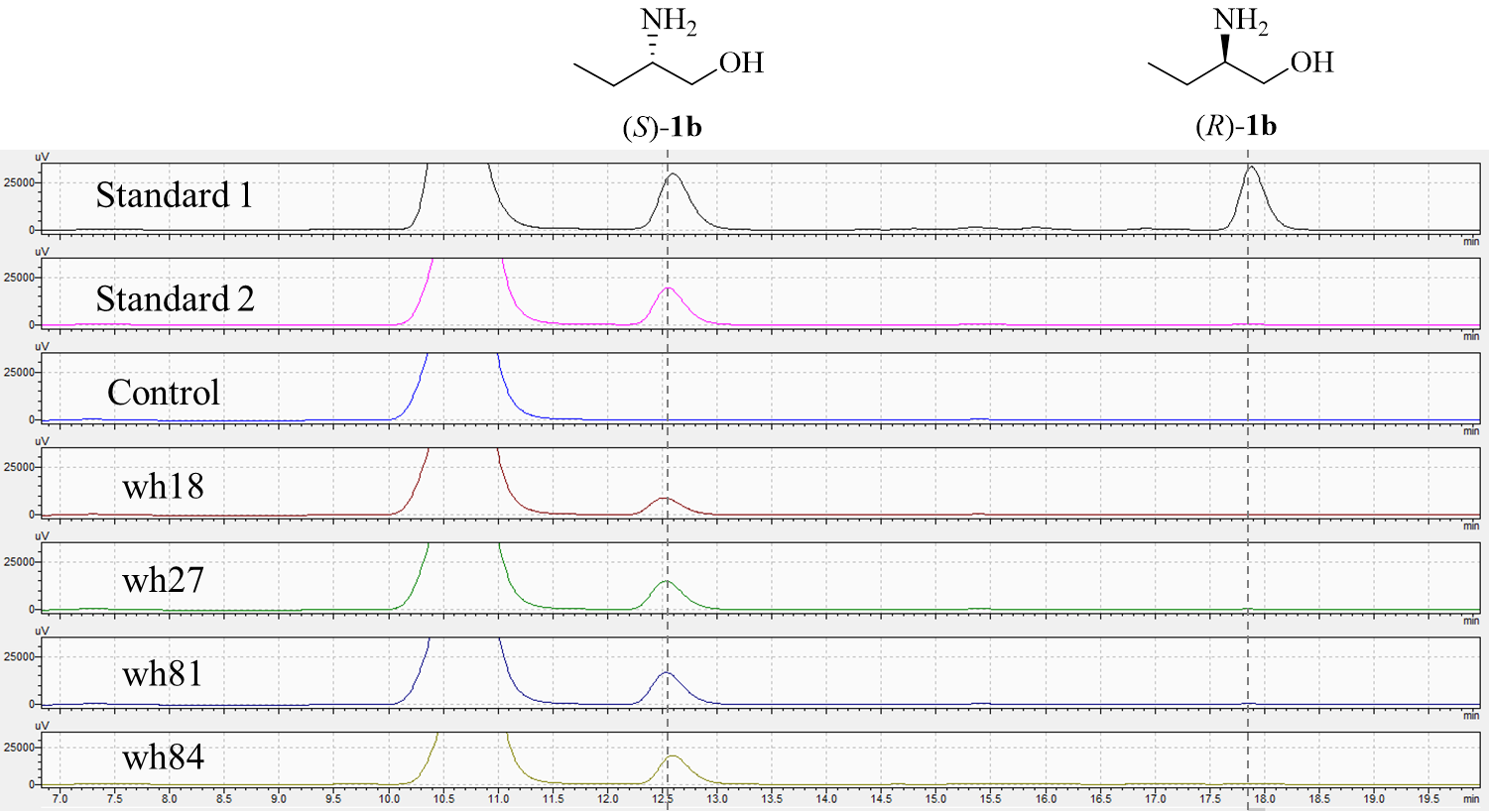


Figure S3. HPLC spectra of (*S*)-1b synthesized from 1a (40 mM) by *Sp*AmDH variants. Standard 1: *rac*-1b, Standard 2: (*S*)-1b.


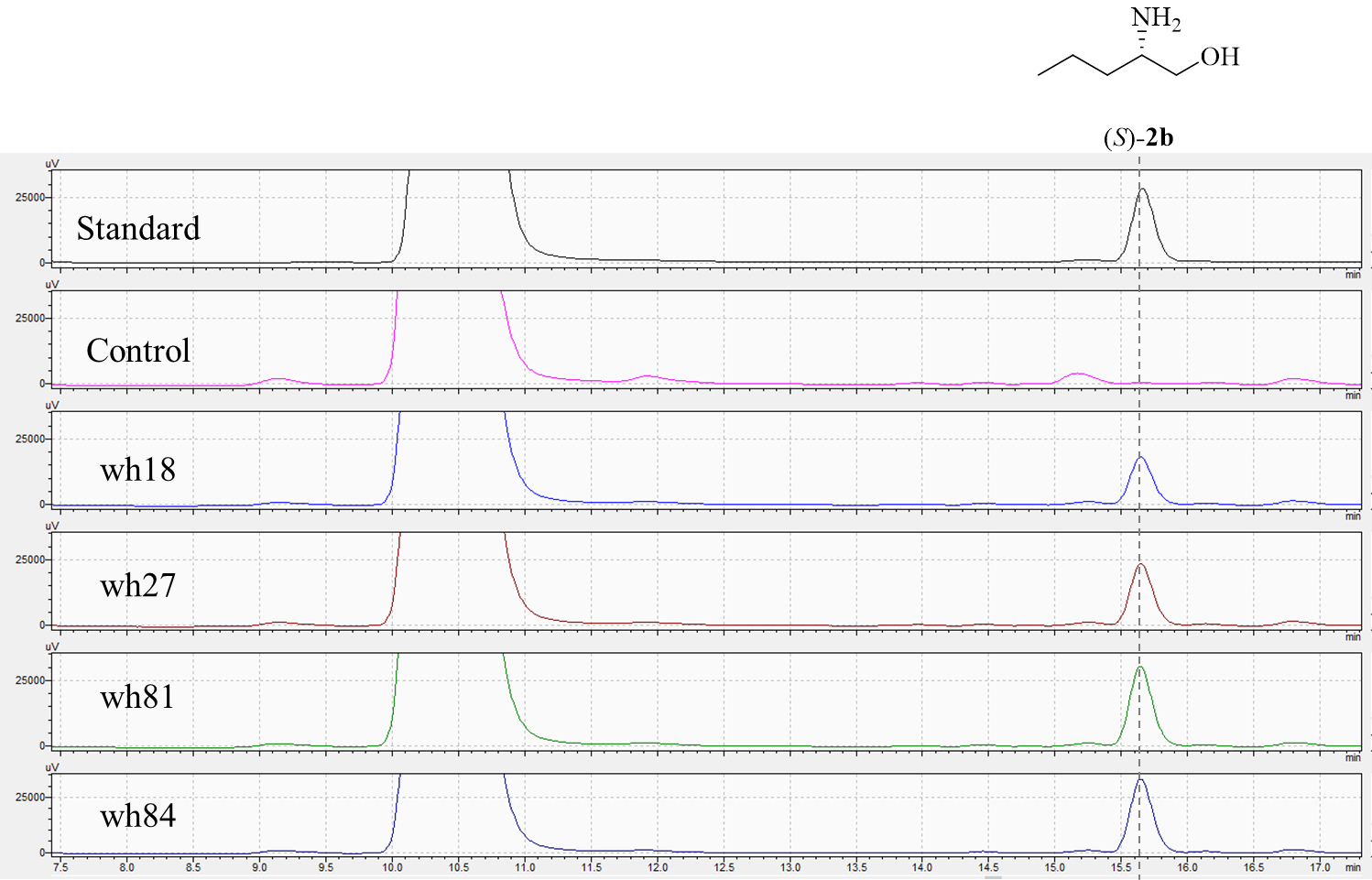


Figure S4. HPLC spectra of (*S*)-2b synthesized from 2a (5 mM) by *Sp*AmDH variants.


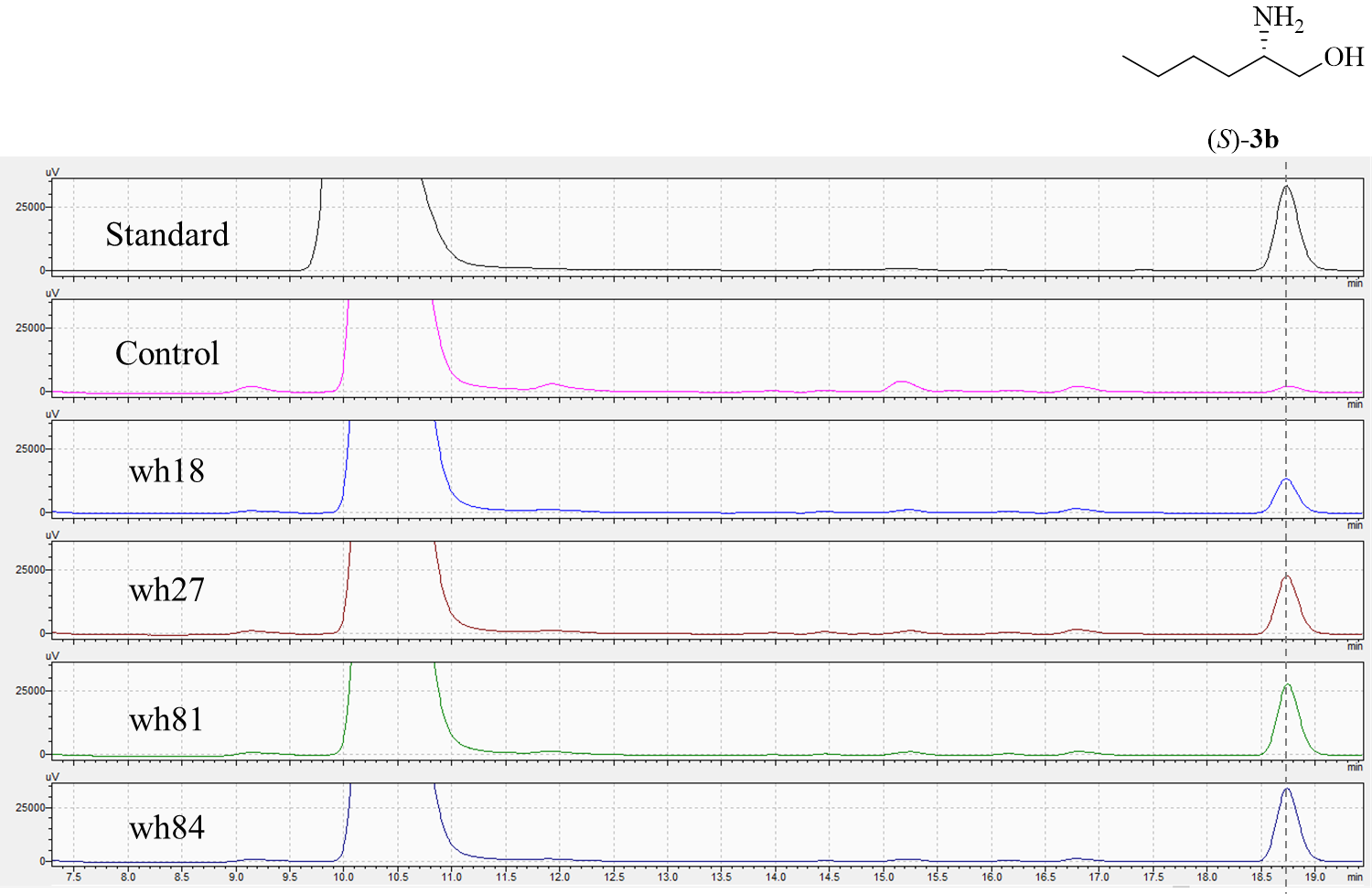


Figure S5. HPLC spectra of (*S*)-3b synthesized from 3a (5 mM) by *Sp*AmDH variants.


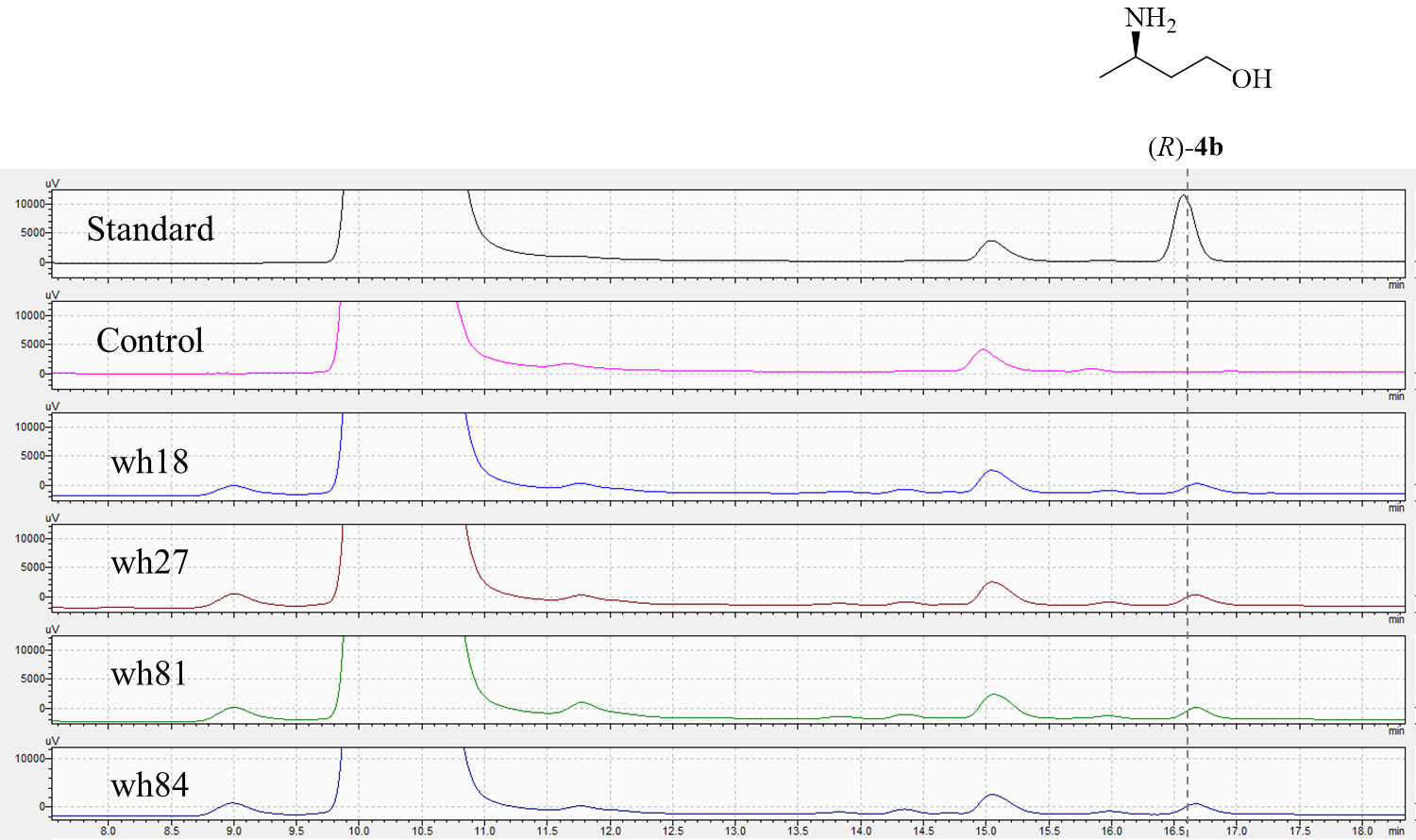


Figure S6. HPLC spectra of (*R*)-4b synthesized from 4a (5 mM) by *Sp*AmDH variants.


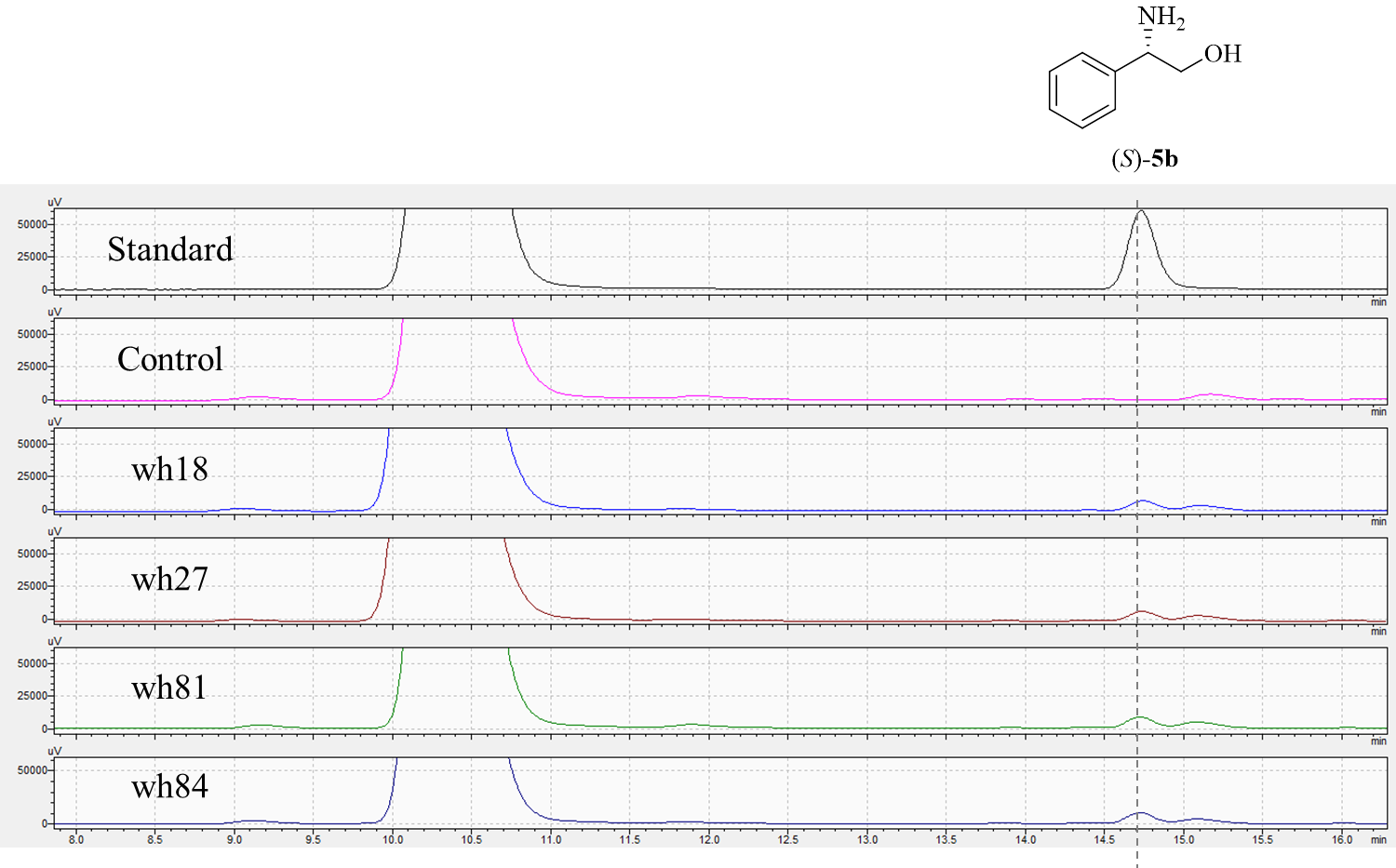


Figure S7. HPLC spectra of (*S*)-5b synthesized from 5a (5 mM) by *Sp*AmDH variants.


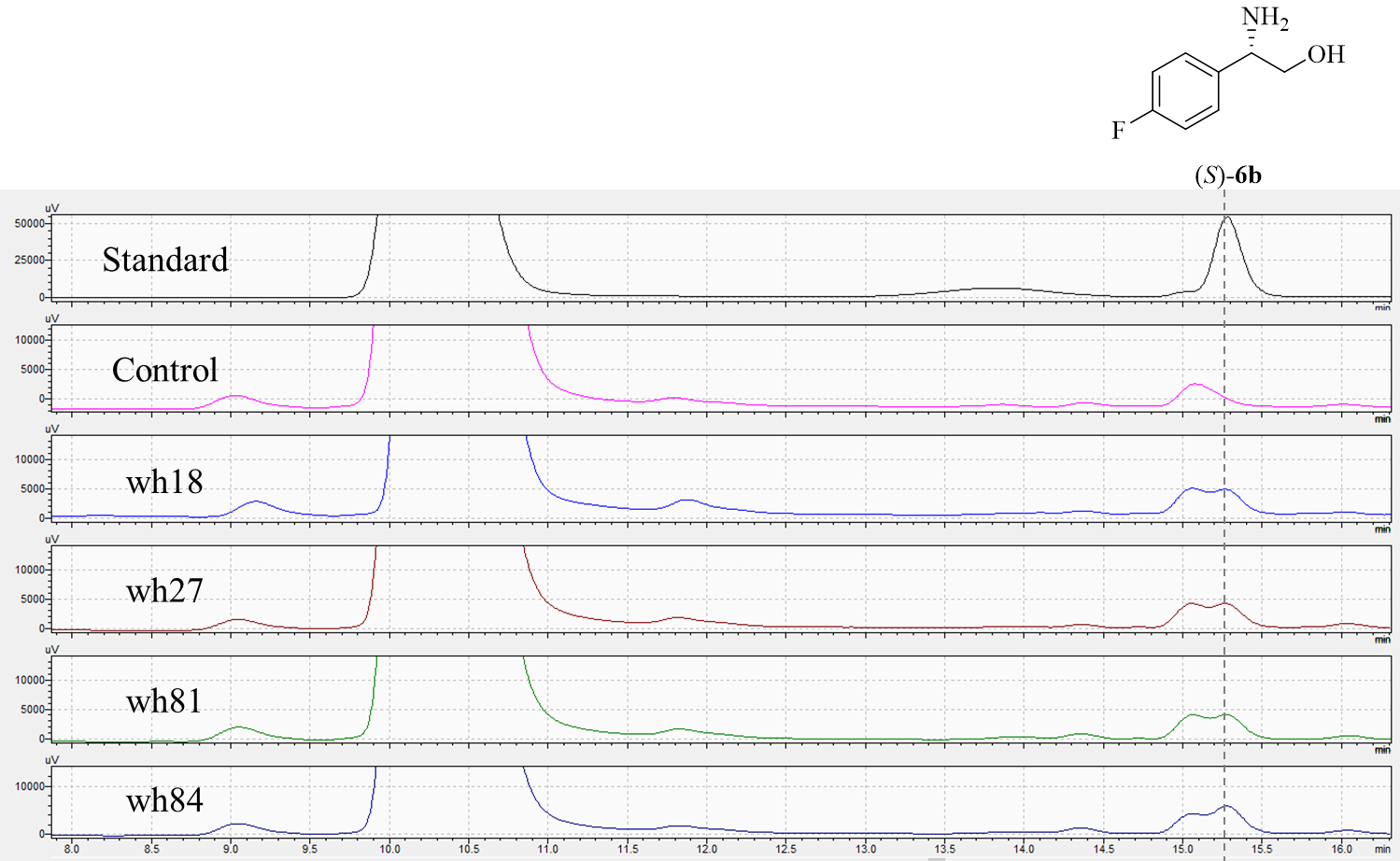


Figure S8. HPLC spectra of (*S*)-6b synthesized from 6a (5 mM) by *Sp*AmDH variants.


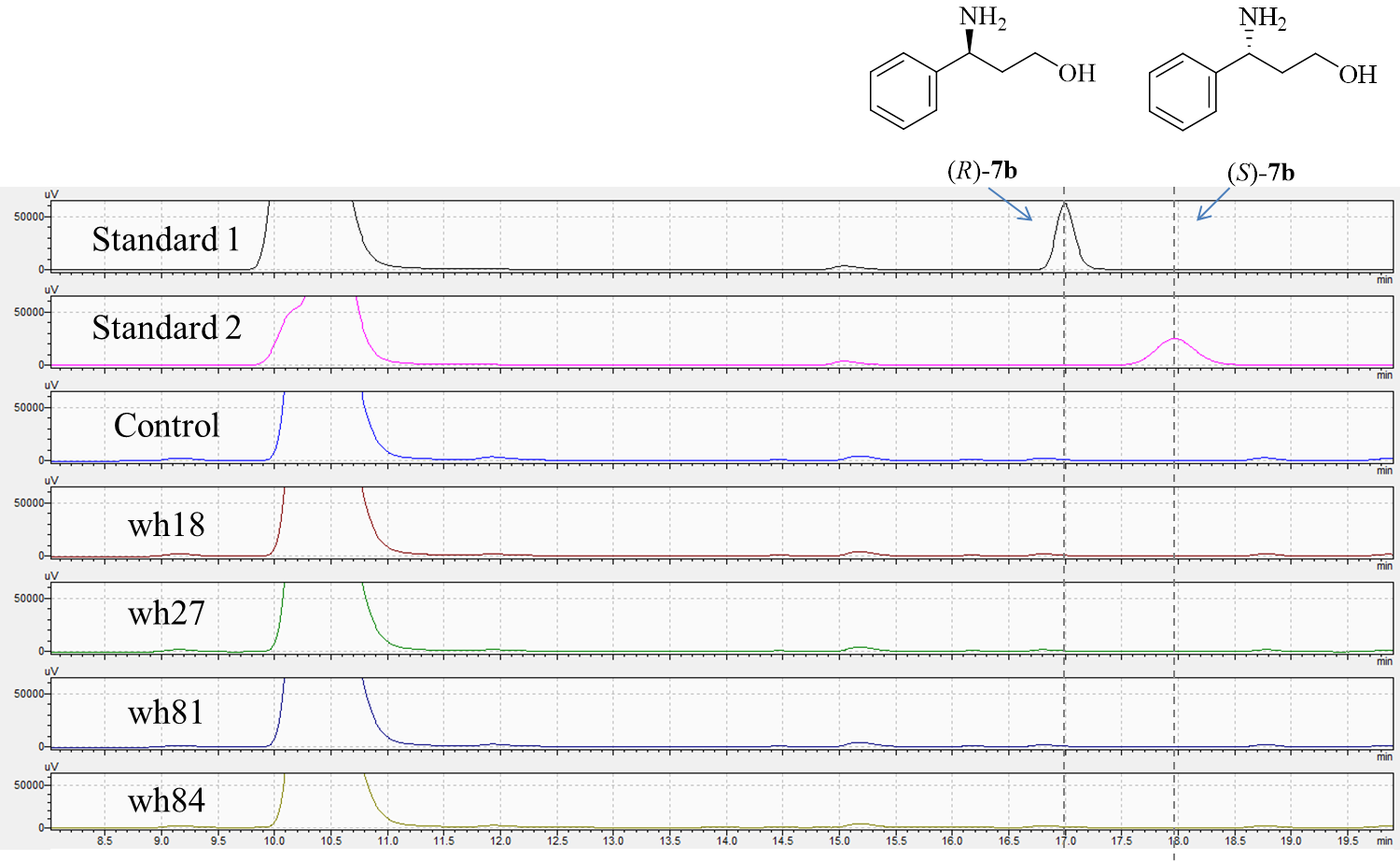


Figure S9. HPLC spectra of 7b synthesized from 7a (5 mM) by *Sp*AmDH variants. Standard 1: (*R*)-7b, Standard 2: (*S*)-7b.

**
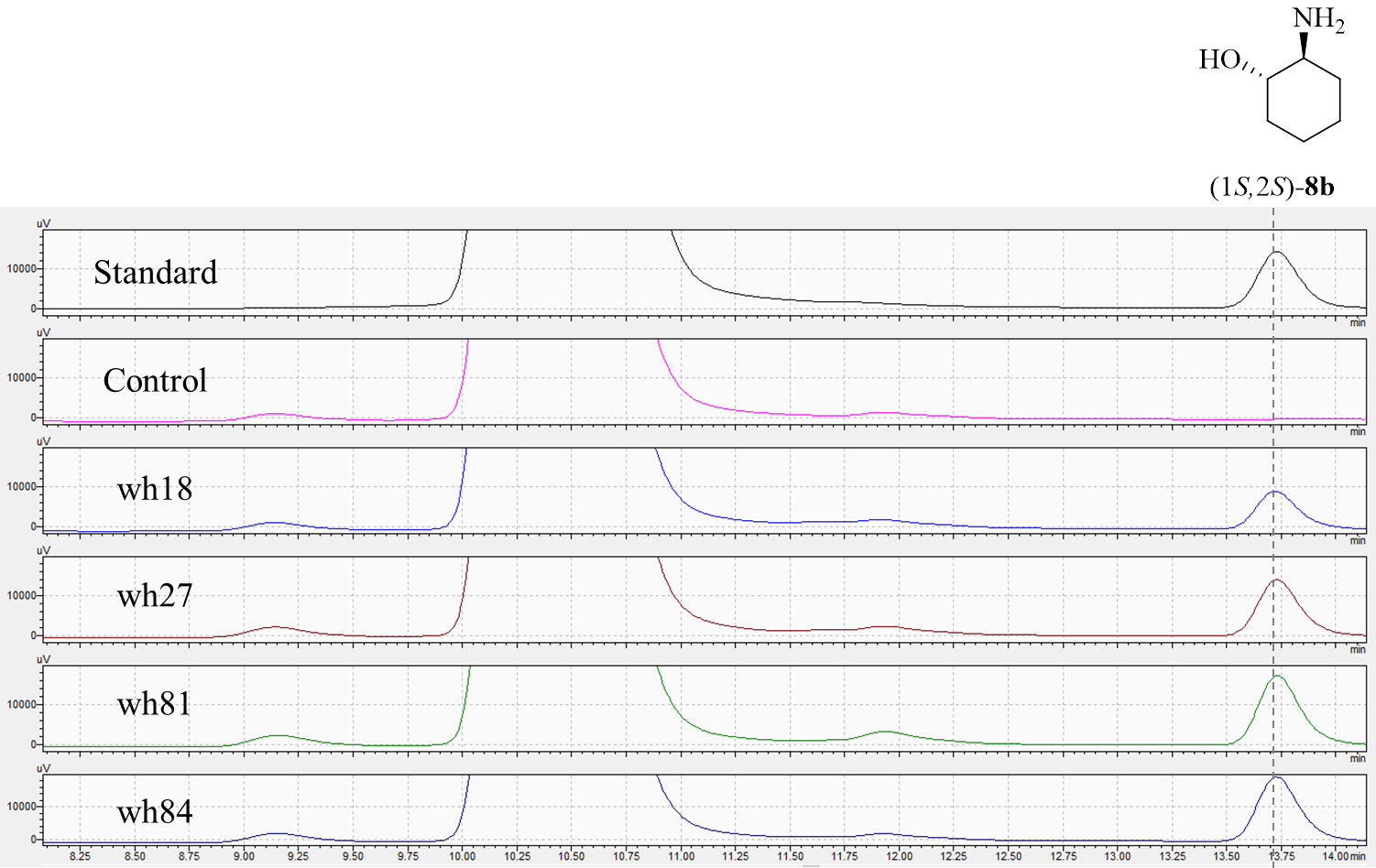
**

Figure S10. HPLC spectra of (1*S*, 2*S*)-8b synthesized from 8a (5 mM) by *Sp*AmDH variants.

**NMR spectra**

[(*S*)-2-aminobutan-1-ol](https://www.chemicalbook.com/Search.aspx?keyword=(S)-(+)-2-Amino-1-butanol%20%3E) (1b)


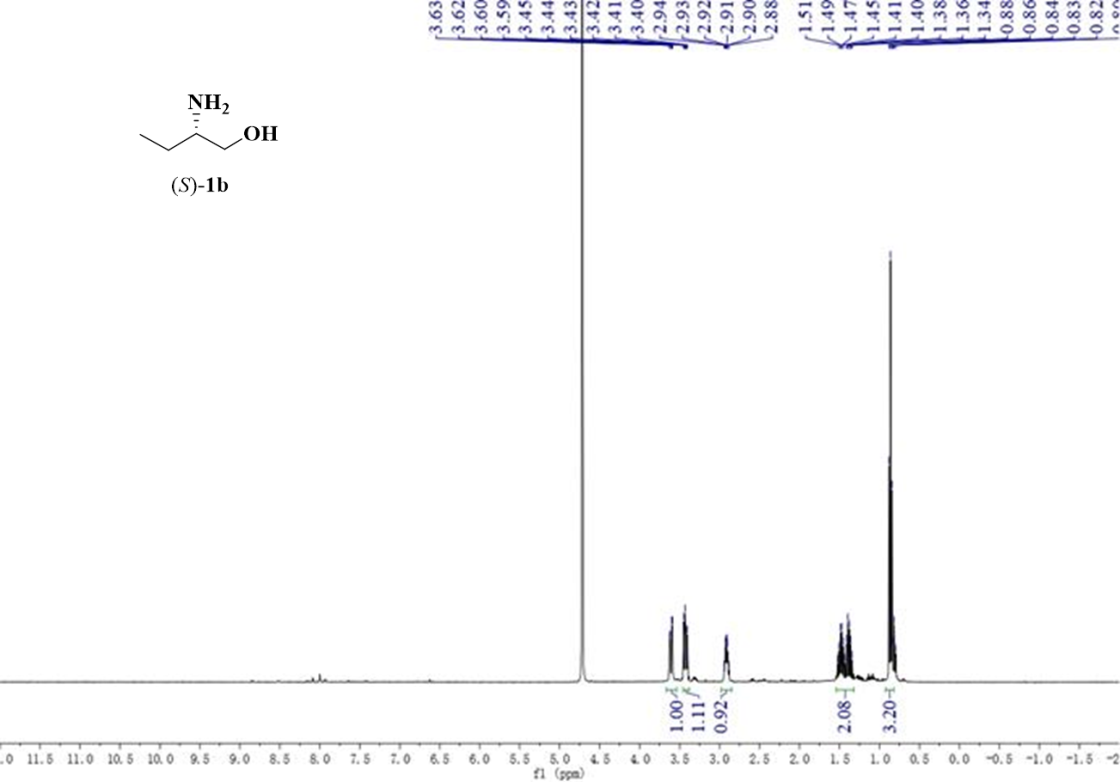

Supplement: Supplementary file 1 [file DataSheet1.docx]
